# ESENA: A Novel Spatiotemporal Event Network Information Approach for Mining Scalp EEG Data

**DOI:** 10.1002/brb3.70426

**Published:** 2025-03-26

**Authors:** Qiwei Dong, Runchen Yang, Xinrui Wang, Zongwen Feng, Chenggan Liu, Shiyu Chen, Yuxi Zhou, Dezhong Yao, Junru Ren, Qi Xu, Li Dong

**Affiliations:** ^1^ Institute of Basic Medical Sciences (IBMS) Chinese Academy of Medical Sciences & Peking Union Medical College (CAMS & PUMC) Beijing China; ^2^ The Clinical Hospital of Chengdu Brain Science Institute, MOE Key Lab for Neuroinformation University of Electronic Science and Technology of China Chengdu China; ^3^ Sichuan Institute for Brain Science and Brain‐Inspired Intelligence Chengdu China; ^4^ Research Unit of NeuroInformation Chinese Academy of Medical Sciences Chengdu China

**Keywords:** EEG, EEG network, spatiotemporal information mining

## Abstract

**Objective:**

Brain activity possesses unique spatiotemporal characteristics. However, few electroencephalogram (EEG) analysis methods were designed to capture these features. Here, we developed a novel approach to mine spatiotemporal information contained in EEG data.

**Methods:**

In this work, a novel approach, named EEG Spatiotemporal Event Network Analysis (ESENA), was proposed to fully capture the complex spatiotemporal patterns of EEG data during rich and complex stimulations. The essence of this method is to map power events onto network nodes and define connections on the basis of the temporal sequence of these events, thereby establishing a spatiotemporal network structure. Next, the performance and feasibility of ESENA were tested using three resting‐state and game‐playing state EEG datasets.

**Results:**

For eyes‐closed resting‐state EEG, specific patterns of spatiotemporal event networks (SENs) were revealed by ESENA for different frequency bands, and the links between SENs were mainly located in regions of rhythmic activity revealed by the relative power spectrum. In the comparison between eyes‐closed and eyes‐open resting‐state EEG, ESENA provided additional important spatiotemporal information in the delta frequency band in the frontal lobe, and in the theta frequency band in the frontoparietal lobes. In the comparison between the game‐playing state and eyes‐closed resting‐state EEG, spatiotemporal information in the delta frequency band in the frontoparietal lobes, the theta frequency band in the parietotemporal lobe and the alpha frequency band in the occipitoparietal lobes was additionally uncovered by ESENA. Moreover, these SENs were correlated with behavioral data.

**Conclusion:**

Our findings demonstrated that the proposed ESENA method is superior to traditional EEG methods in discovering spatiotemporal patterns from EEG data and has the potential to become an important tool providing deeper insights into the brain's complex networks.

## Graphical abstract

A novel method, named ESENA, effectively captures complex spatiotemporal patterns in EEG data across different brain regions. Tested on multiple EEG datasets, ESENA revealed critical spatiotemporal information not detected by traditional methods, providing deeper insights into brain network dynamics during various cognitive states.

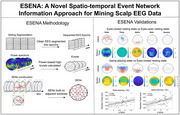

## 1 Introduction

The execution of brain functions requires tight coordination among various areas; this reflects the complex interdependencies of functional patterns, especially in the context of spatiotemporal information processing (Stark et al. 2008). The complex brain functions originate from localized activation of specific regions, subsequently evolving through close coordination between these areas into comprehensive spatiotemporal network processes (Bressler and Kelso 2016). As the importance of spatiotemporal information in brain functions has become widely recognized (Northoff and Scalabrini 2021), a number of researches have been conducted (Dong, Luo, et al. 2014; Iakovidou et al. 2013; Atluri et al. 2014), highlighting its critical role in enhancing our understanding of brain functionality and various neurological conditions.

EEG is a noninvasive, portable, cost‐effective, and high‐temporal resolution technology for detecting brain activity (Z. Zhang 2019). It is used extensively for recording brain electrical signals to aid neurological disease diagnosis, as well as in brain–computer interfaces and brain apparatus communication (Yao et al. 2022). As a typical nonstationary signal, EEG provides a high‐temporal‐resolution imaging of the brain's spatiotemporal processes (Lo et al. 2011). In EEG analysis, data can be analyzed in temporal, spatial, and spatiotemporal contexts. (1) In the time domain, time‐frequency (TF) analysis is a conventional method for analyzing nonstationary signals, such as EEG data, and it excels in depicting dynamic changes in frequency, power, and phase features over time (Morales and Bowers 2022). This analysis provides an approach for quantifying signal power at specific TF points (e.g., short‐time Fourier transform and continuous wavelet transform) or decomposing the signal on the basis of unique TF characteristics (e.g., discrete wavelet transform and empirical mode decomposition) (Z. Zhang 2019). The main advantages of TF analysis are its interpretability and comprehensive tracking of oscillatory brain activities, exemplified by its use in event‐related intertrial coherence analysis to efficiently discern phase‐ or time‐locked responses to stimuli (Morales and Bowers 2022). Other time‐domain methods include nonlinear dynamical analyses, such as Lempel–Ziv complexity‐ and entropy‐based methodologies (Z. Zhang 2019). The abovementioned methods collectively advanced our understanding of brain functionality and state through EEG time series analysis. (2) Regarding spatial information extraction, various techniques have been proposed. For example, EEG power spectral analysis is a conventional cut‐in point that can reveal the degree of brain activity on the scalp (Chikhi et al. 2022). The EEG source localization method can specify neural activity by analyzing EEG potential distributions and inversing scalp surface electrical signals to the cerebral cortex, offering insights into the neural underpinnings of cognitive and emotional processes (Michel and Brunet 2019). However, considering that EEG is a biological signal providing substantial spatiotemporal information, analyzing EEG data only in the spatial or temporal domain is not sufficient (Cole and Voytek 2017). It is essential to approach the analysis from an integrated spatiotemporal perspective. Therefore, spatiotemporal methods for EEG analysis were proposed. (3) In the spatiotemporal domain, microstate analysis is a conventional technique that focuses on the segmentation of brain electrical activity into brief, quasi‐stable topographies, reflecting the spatiotemporal organization of neural processes and capturing the dynamic interplay between spatial patterns over time (Tarailis et al. 2023). Simultaneous EEG‐fMRI fusion is another method for uncovering spatiotemporal information, effectively exploiting the high spatial detail of fMRI and the temporal superiority of EEG to offer a novel view of brain functions (Dong, Gong, et al. 2014). Recently, a new EEG analysis method based on “cascading high‐amplitude bursts in neural activity” was also proposed to reveal sequential patterns of intense neural activity that unfold over time, in turn revealing the complex spatiotemporal dynamics of brain interactions, including in pathological conditions (Bansal et al. 2021). However, challenges remain for spatiotemporal EEG methods. For example, previous studies have primarily focused on exploring the activities associated with specific neural processes or occurring in localized brain regions, with an emphasis on the dynamics of neural synchronization within certain areas. While these studies have advanced our understanding of spatiotemporal patterns in brain electrophysiology, they often overlooked the holistic characteristics of the brain as a complex network. Furthermore, a significant challenge in the extraction and mining of spatiotemporal information is dealing with the high dimensionality of the data and analyzing temporal and spatial information at the same time.

Networks provide a comprehensive framework for neuroscience research, particularly by depicting sub‐manifolds within dimensional spaces and capturing the dynamic and topological aspects of brain activity (Zou et al. 2019). This includes revealing the brain's hierarchical structures, as well as global or local patterns that are independent of data distribution. This approach not only emphasizes the importance of a holistic view but also exhibits unique advantages in the spatiotemporal information mining (STIM) (Shekhar et al. 2015) of EEG data. It can uncover long‐term dynamic interactions and patterns of connectivity between different brain regions and frequency bands in multidimensional data (Bassett and Sporns 2017). Functional connectivity (FC), as inferred from time series data, forms the basis of networks that emphasize the temporal synergy of local and distant brain regions. This network‐centric view is fundamental to various neural information techniques such as EEG and fMRI (Sporns 2013) and reveals the dynamic interplay of brain functions across spatial and temporal scales (Lang et al. 2012). Traditional FC analyses predominantly emphasize the spatial dimension of FC patterns, resulting in the loss of critical temporal information (Lamoš et al. 2018). In contrast, recent STIM methods, such as dynamic functional connectivity (DFC) analysis (C. Zhang et al. 2018), the four‐dimensional (spatiotemporal) Consistency of local neural Activities (FOCA) (Dong et al. 2016), and group independent component analysis (Calhoun et al. 2009), which are primarily aimed at fMRI rather than EEG data, analyze time‐series data to uncover spatiotemporal FC patterns within brain regions. In fact, the functionality of the brain relies on dynamic interactions among multiple cerebral regions, where several activation events occur synchronously across different regions over time (McAfee et al. 2021). Therefore, because EEG power in specific frequency bands often varies under different cognitive or physiological states, it may reflect events occurring during EEG recording (Hamada et al. 2023). Moreover, concurrent activation at adjacent temporal points might indicate FC between different brain areas, forming a functional network (or a subset thereof) that stabilizes over time such that it is characterized by a recurring pattern (Iakovidou et al. 2013; Ferreira et al. 2020; Smith et al. 2012).

On the basis of the above assumptions, in this work, we propose a novel approach, named EEG Spatiotemporal Event Network Analysis (ESENA), to capture the complex spatiotemporal patterns of EEG data under conditions of rich and complex stimulation (i.e., while playing games [game‐playing state]). The essence of this method is to map power events onto network nodes and define connections on the basis of the temporal sequence of the events, thereby establishing a spatiotemporal network structure. As the event sequence unfolds, this structure retains consistently appearing patterns. Next, the performance of ESENA was tested on the basis of various parameters, including epoch length, event threshold, and data length, using resting‐state EEG data. Subsequently, an EEG dataset containing both eyes‐open resting state (EC) and eyes‐closed resting state (EO) data was used to validate the effectiveness of this analytical method. Finally, an EEG dataset acquired during the game‐playing state was investigated to test the feasibility of using ESENA to reveal brain functional changes in practical studies.

## 2 Material and Methods

### 2.1 Participants

For EEG Dataset 1, EC EEG data were collected from 66 healthy individuals (age range: 18–21 years; mean ± standard deviation age = 19.79 ± 0.88 years; all right‐handed). For Dataset 2, originating from a different experiment than Dataset 1, EEG data were collected from 25 healthy participants (age range: 20–30 years; mean age = 24.05 ± 1.90 years; all right‐handed) using the same selection criteria applied to obtain Dataset 1. Both EC and EO recordings were collected from each participant in Dataset 2. Dataset 3 comprised 55 healthy individuals (age range: 18–21 years; mean ± standard deviation age = 19.66 ± 0.87 years; all right‐handed), whose most effective data length for both EC and game‐playing state exceeded 90 s. The game‐playing EEG recordings were captured while the participants, with their heads stabilized, were playing the game “League of Legends” (Riot Games). Before EEG data collection, participants also completed attention span and distributed spatial attention tasks, details of which can be found online (Zhou et al. 2020). The experiments were approved by the Ethics Committee of the Life Science and Technology Department, University of Electronic Science and Technology (Chengdu, China). All participants gave written informed consent.

### 2.2 EEG Acquisition and Preprocessing

Datasets 1 and 3 were collected using the actiCHamp Plus and 32‐channel actiCap slim active electrodes (Brain Products GmbH, Gilching, Germany), and Database 2 was collected using 64‐channel actiCap slim active electrodes (Brain Products GmbH). The electrode distribution adhered to the 10–20 system, and collected data underwent online band‐pass filtering (0.01–100 Hz). A 32‐channel EEG recording system was employed, incorporating a reference electrode and a sampling rate of 1,000 Hz. Figure S1a shows the channel distribution. The 64‐channel EEG system included two electrooculography electrodes and the original recording reference electrode, with a sampling rate of 500 Hz. Figure S1b shows the channel distribution for this system.

All data were assessed for quality and preprocessed using the preprocessing pipeline of the WeBrain platform (Dong, Li, et al. 2021). The main procedures were as follows: (1) a quality assessment method was used to detect bad channels with different types of artifacts (constant or not a number/infinite signals, unusually high or low amplitudes, high‐ or power frequency noises, and low‐correlation signals) (Zhao et al. 2023); (2) EEG signals were subjected to bandpass filtering in the range of 1–60 Hz, with the addition of a 50‐Hz notch filter to eliminate power line interference; (3) the independent component analysis‐based MARA algorithm was used to removal artifacts; and (4) the reference electrode standardization interpolation technique was applied to interpolate the bad channels (Dong, Zhao, et al. 2021) and transform the reference electrode into an idealized zero potential point at infinity (Dezhong 2001). Finally, further analyses were conducted on uncontaminated EEG data.

### 2.3 Spatiotemporal Event Network Construction

**FIGURE 1:**
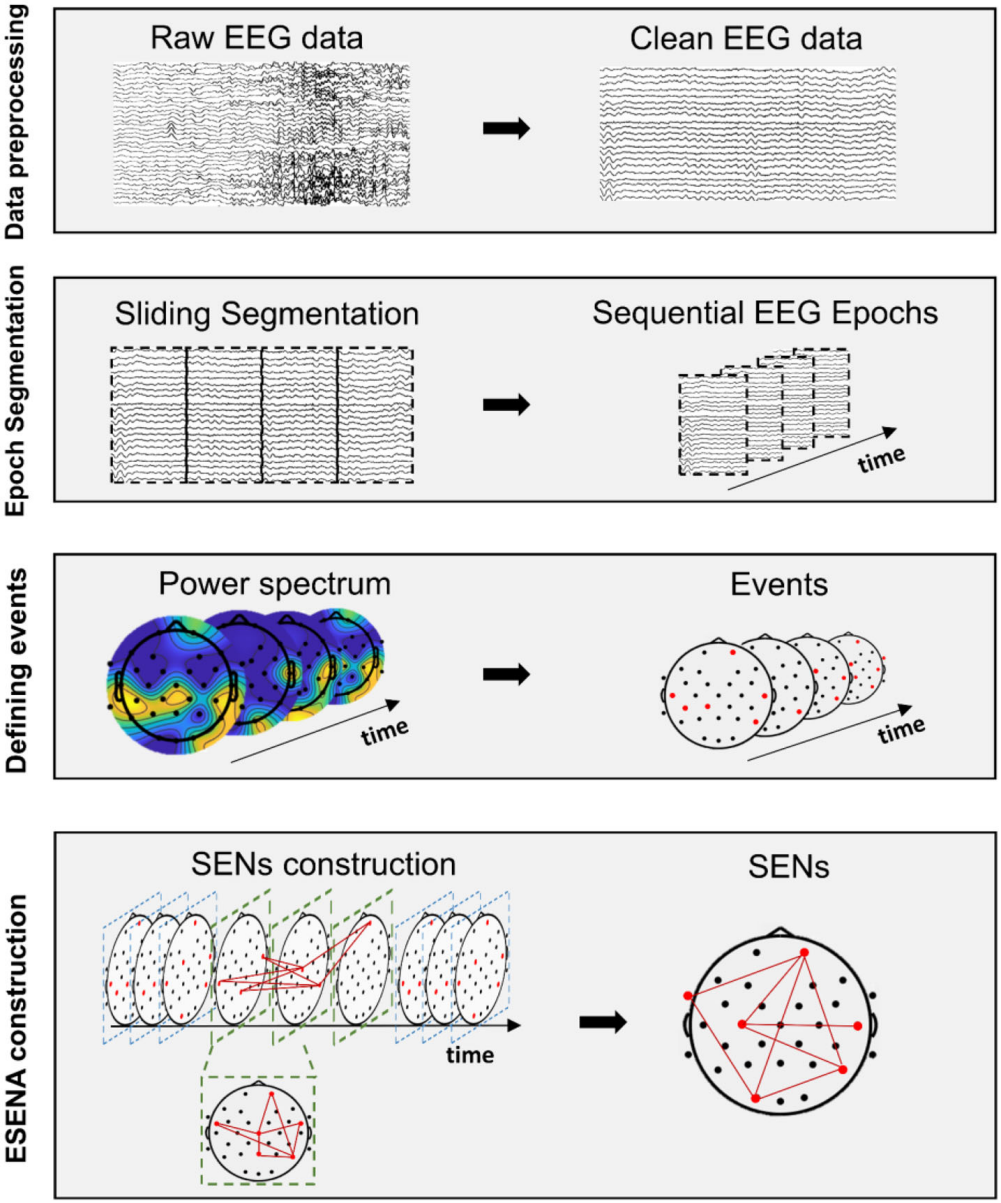
Construction of SENs. (1) Preprocessing the raw EEG data; (2) segmenting the clean EEG data into nonoverlapping time‐series epochs using sliding windows; (3) calculating the relative power spectrum for each epoch in the cleaned EEG data and selecting events; (4) connecting events occurring in adjacent epochs and retaining frequently occurring links. ESENA, EEG Spatiotemporal Event Network Analysis; SENs, spatiotemporal event networks.

We have developed an approach to convert brain EEG data into spatiotemporal event networks (SENs) (Ferreira et al. 2020), wherein links represent chronological events occurring within the brain, as illustrated in Figure 1. This approach operates on the basis of a time‐ordered and clean spatiotemporal EEG dataset (*X* = {*e*_11_ *, e*_12_ *, e*_13_ *, …, e*_LT_}), where an event *e*_ab_ = (*l*_a_, *t*_b_) is denoted by its spatial coordinates (EEG channel locations; *l*_a_ = [*x*_a_ *, y*_a_ *, z*_a_] ∈ {*l*_1_ *, l*_2_ *, l*_3_ *, …, l*_L_}) and temporal dimension *t*_b_ ∈ {*t*_1_ *, t*_2_ *, t*_3_ *, …, t*_T_} (time series). The construction of the SENs involves a four‐stage process. First, the brain is segmented into distinct nodes on the basis of designated regions of interest, resulting in an EEG set *L* = {*l*_1_ *, l*_2_ *, l*_3_ *, …, l*_L_}, which corresponds to the array of channels in the EEG recording system. Notably, the nodes set *L* is EEG electrodes based on the 10–20 system in this study. Then, a time frame Δ*t* is defined, segmenting the spatiotemporal data at equal intervals of Δ*t*. Because of certain connectivity prerequisites, it is essential that *T* is at least 2Δ*t*. The third stage involves defining an event *e*_n_. In our methodology, events are identified by high‐energy bursts. To achieve this, we calculate the relative power (*P*_t_ = {*p*_1t_ *, p*_2t_ *, p*_3t_ *, …, p*_Lt_}) for all nodes within each time segment and set a threshold (*Th* = {*th*_1_ *, th*_2_ *, th*_3_ *, …, th*_T_}) for event occurrence within every segment. Nodes with power exceeding the threshold within a time window are designated as events occurring in that particular window. In the final stage, events from consecutive windows are interconnected. Specifically, if two consecutive events *e*_ib_ and *e*_j(b + Δt)_ occur in adjacent time windows (to simplify, we did not establish links between events within the same time window, but this does not hinder the generalizability of connections within the same window in ESENA), a link is established between nodes *l*_i_ and *l*_j_, where *w*_ij(b + Δt)_ = *w*_ij(b)_ + 1(*I *≠ *j*, because when *i *= *j* ESENA primarily extracts events occurring consecutively across regions; events within the same region are akin to results from conventional power spectra). These connections can be either directed or undirected; however, because of the focus on capturing the entirety of sequential events between nodes, undirected links were preferred in this study.

The construction process proceeds from the events in the initial time window to those in the final window. The link weight *w*_ij_ between nodes *l*_i_ and *l*_j_ signifies the aggregation of successive events. The weight of a link illustrates the likelihood of its occurrence: a lower weight hints at incidental emergence, whereas a higher weight suggests a more consistent and predictable emergence. The weight *w*_ij_ is adjusted using Equation 1:

(1) $$cw_{\mathrm{ij}}=\frac{w_{\mathrm{ij}}}{w_{\max}}$$

where *w*_ij_ is the uncorrected weight, *w*_max_ is the maximum weight in the entire time window, defined as *w*_max_ = max(*w*_ab_), with a,b∈{1,2,…,*L*}, and *cw*_ij_ is the adjusted weight. After adjustments, links that pass a one‐sample *t*‐test threshold (false discovery rate [FDR] < 0.05) and appear in > 60% of participants in the dataset are retained.

In addition, the definition of an event is adaptable and can be modified according to specific requirements. In this study, we define an event on the basis of elevated EEG power at specific spatiotemporal points, as the power spectrum is one of the most traditional cut‐in points for EEG studies. Subsequent analyses focused on standard EEG frequency bands: delta (1–4 Hz), theta (4–8 Hz), alpha (8–12.5 Hz), beta (12.5–30 Hz), and gamma (30–60 Hz) (de la Salle et al. 2016). Meanwhile, two‐tailed one‐sample *t*‐tests were carried out on the sample data of SENs and the relative power results obtained in each state to determine whether these data significantly deviated from the hypothesized zero mean. For the evaluation of differences between states, the two‐tailed paired *t*‐test was performed. All analyses were corrected for multiple comparisons using the FDR method. Additionally, Pearson correlation coefficients were calculated to explore the relations between SENs for game‐playing data and behavioral performance (attention span and distributed spatial attention tasks). To enhance the clarity of the ESENA results, the connections between different brain regions were calculated as a proportion of the total connections in each frequency band, with the distribution of electrodes determined according to relevant literature (Guo et al. 2022).

### 2.4 Parameter Settings

**FIGURE 2:**
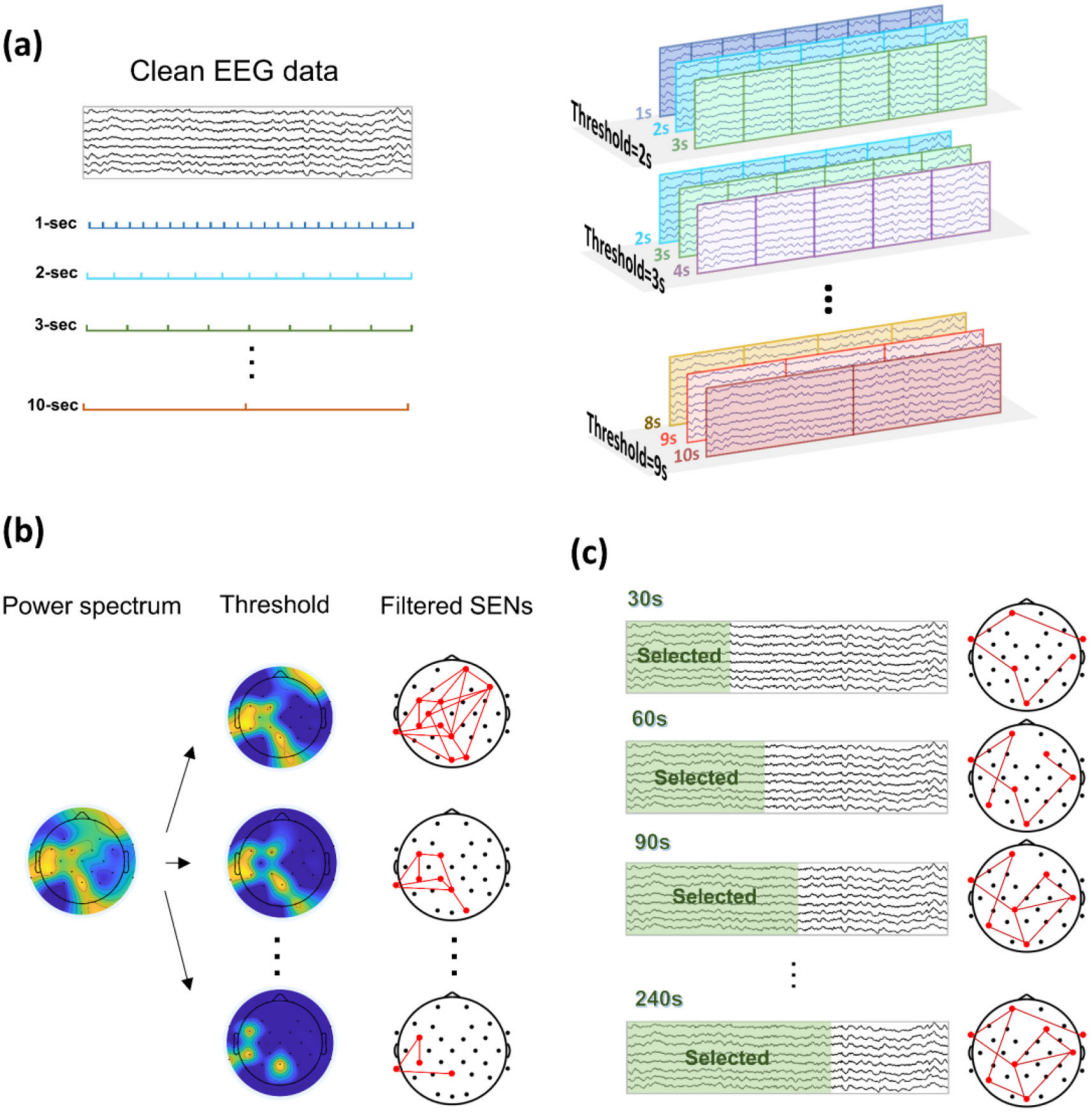
ESENA parameters. (a) Segmenting the clean EEG data into epochs of varying lengths and calculating the intraclass coefficients for the relative power spectrum across different epoch length groups. (b) Using the mean + 0.5–1.5 standard deviation power spectrum as the threshold to filter events and construct SENs. (c) Selecting data lengths of 30–240 s to construct SENs. ESENA, EEG Spatiotemporal Event Network Analysis; SENs, spatiotemporal event networks.

In parameter selection, ensuring the stability of results is crucial. Stable results not only guarantee the consistency and reproducibility of the spatiotemporal patterns discovered but also indicate that these patterns are not merely the product of random fluctuations or noise but rather reflect meaningful and reliable features of the brain's functional network (Ruiz et al. 2009). As shown in Figure 2a (left), the data from Dataset 1 underwent a fast Fourier transform and were segmented into 1–10‐s epochs after preprocessing, which helped obtain more stable signal features due to the nonstationary nature of EEG signals, allowing for more accurate analysis of rapid changes in brain activity. The relative power of these frequency bands was then calculated using the power calculation tool of the WeBrain toolbox (Dong, Li, et al. 2021). The intraclass correlation coefficient (ICC) was calculated for each group using a two‐way random effects model (K. Zhang, Shi, et al. 2021), where three adjacent epoch lengths were grouped as shown in Figure 2a (right). As shown in Figure 2b, after computing the relative power spectrum for each electrode across frequency bands, the event threshold was established. This threshold was set as the mean + 0.5–1.5 standard deviation, which is a common and well‐established method in statistical analysis (Niknazar et al. 2020; Arviv et al. 2015). Subsequently, singular value decomposition (SVD) was applied to reduce the dimensionality of each edge for every participant, allowing the extraction of the first principal component (PC) from all edges. Dimensionality reduction facilitates the isolation of the most informative components, which capture a large portion of the variance in the data while filtering out less significant components that may be influenced by noise or individual differences. Finally, the first PC of each edge was then averaged across all frequency bands, yielding a more robust SEN representation. The same methodological approach was applied to select the data length threshold (range: 30–240 s), as depicted in Figure 2c.

## 3 Results

In this study, Database1, a 32‐channel eyes‐closed resting‐state dataset, was initially used to determine the key parameters of ESENA, including epoch length, event threshold, and data length. Following this, Database2, a 64‐channel dataset featuring both EC and EO paradigms, and Database3, a 32‐channel dataset incorporating both EC and game‐playing state paradigms, were utilized to further validate the effectiveness of ESENA across different electrode distributions and task conditions, such as EC, EO, and game‐playing state.

### 3.1 ESENA Parameter Settings

**FIGURE 3:**
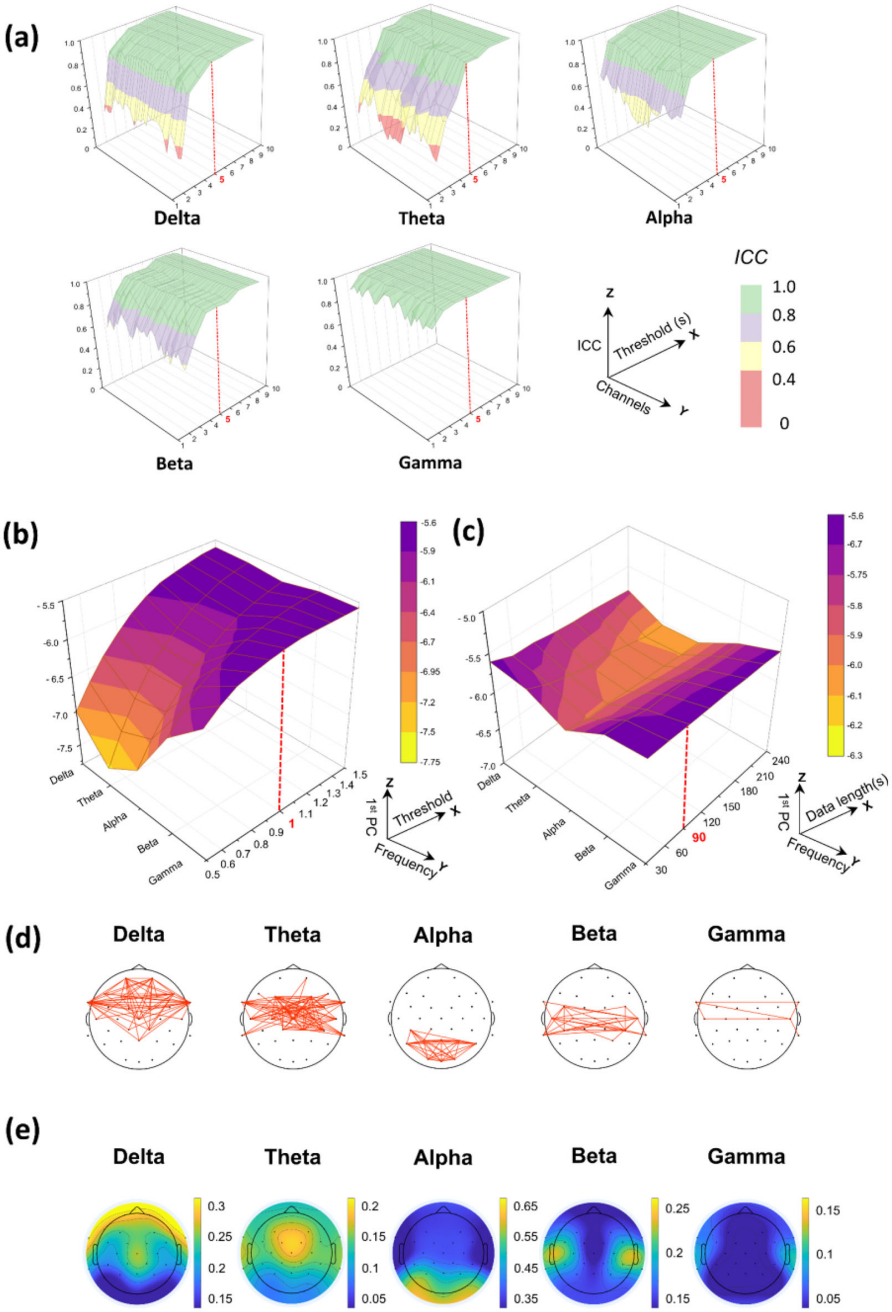
Parameter results. (a) Epoch length selection. The ICC values are mapped over varying epoch durations ranging from 2 to 8 s, represented on the *X*‐axis. The *Z*‐axis corresponds to ICC values, where values < 0.4 indicate poor stability and those > 0.8 signify high stability. (b)–(c) Event threshold and data length selection. These results depict the relationship between threshold values and data length (*X*‐axis) according to the EEG frequency band (*Y*‐axis). The *Z*‐axis corresponds to the first PC. (d)–(e) Spatiotemporal event networks and relative power spectrum of Database 1 (one‐sample *t*‐test, FDR < 0.05). ICC, intraclass correlation coefficient; PC, principal component.

Figure 3a demonstrates that the delta, theta, and alpha frequency bands remained stable after 5 s, while the beta and gamma frequency bands stabilized after 4 and 3 s, respectively. High and stable ICCs were obtained at the 5‐s epoch across all bands.

Therefore, the epoch length was set to 5 s for ESENA analysis in this study. As shown in Figure 3b, for the delta, theta, and alpha bands, the first PCs quickly changed from mean + 0.5 to +1 standard deviation and then remained stable. In contrast, beta and gamma bands changed more gradually but also stabilized after reaching the mean + 1 standard deviation. Therefore, the mean + 1 standard deviation was selected as the event threshold. Figure 3c depicts the variation in the first PC according to data length. Except for the theta band, all frequency bands’ data lengths stabilized after 90 s; the theta band showed a relatively rapid decline between 30 and 90 s, slowing down and remaining largely stable thereafter. Therefore, 90 s was chosen as the data length threshold.

### 3.2 Spatiotemporal Event Networks

#### 3.2.1 Eyes‐Open Resting State

For Dataset 1's EC subset, Figure 3d and Table 1 display SENs and their connection counts percentage, and Figure 3e shows the relative power results. In the delta and theta bands, over 90% of the connections were associated with the frontal lobe, with the highest proportion of connections occurring within the frontal region. For the alpha band, all connections were related to either the occipital or parietal lobes. In the beta and gamma bands, the vast majority of connections were associated with the temporal lobe. The results for the relative power spectrum showed that the links between SENs were largely located in regions of rhythmic activity during the EC.

**TABLE 1:** Percentage of connection counts in Figure 3d.

	F‐F	F‐P	F‐O	F‐T	P‐P	P‐O	P‐T	O‐O	O‐T	T‐T
Delta	54.9%	9.8%	—	27.5%	—	—	3.9%	—	—	3.9%
Theta	39.3%	23.0%	—	31.2%	1.6%	—	1.6%	—	—	3.3%
Alpha	—	—	—	—	25.7%	42.8%	14.3%	8.6%	8.6%	—
Beta	—	12.5%	—	20.8%	8.4%	—	37.5%	—	—	20.8%
Gamma	—	—	—	33.3%	—	—	16.7%	—	—	50.0%

Abbreviations: F‐F, fronto‐frontal; F‐O, fronto‐occipital; F‐P, fronto ‐ parietal; F‐T, fronto ‐ temporal; NegT, Negative *t*‐test result; O‐O, occipito‐occipital; O‐T, occipito ‐ temporal; P‐O, parieto‐occipital; PosT, Positive *t*‐test result; P‐P, parieto‐parietal; P‐T, parieto ‐ temporal; T‐T, temporo‐temporal.

#### 3.2.2 EC vs. EO

**FIGURE 4:**
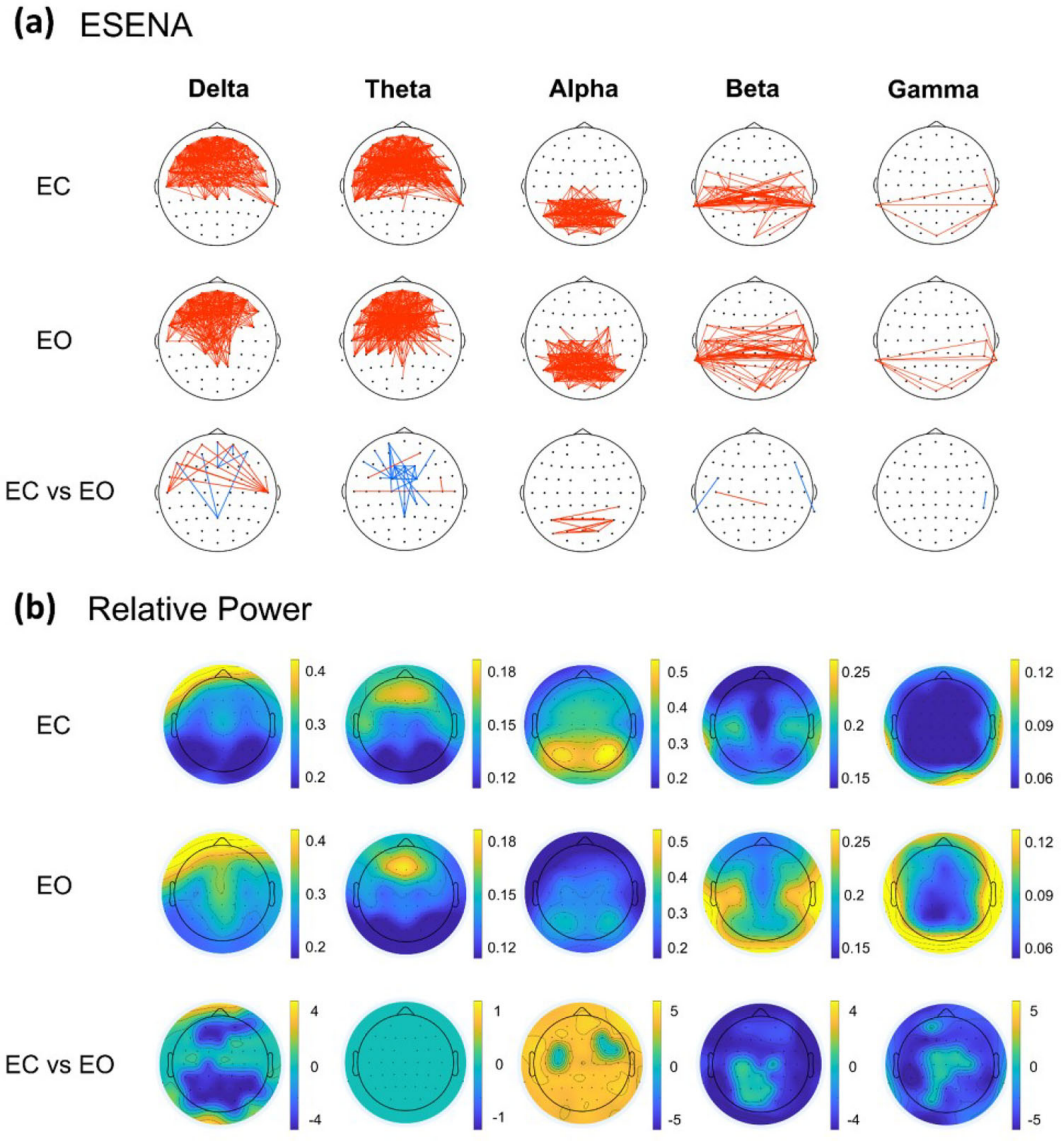
ESENA and relative power results for the resting state. (a) Spatiotemporal event networks of the EC, EO (one‐sample *t*‐test, FDR < 0.05), and EC versus EO (paired *t*‐test, *p* < 0.01; red: EC > EO, blue: EC < EO). (b) The relative power of the EC, EO (one‐sample *t*‐test, FDR < 0.05), and EC versus EO (paired *t*‐test, *p* < 0.01). ESENA, EEG Spatiotemporal Event Network Analysis; EC, eyes‐closed resting state; EO, eyes‐open resting state.

Figure 4a and Table 2 illustrate the ESENA results, while Figure 4b presents the relative power spectrum for the EC and EO subsets of Dataset 2. In the delta and theta bands, over 90% of connections during EC were primarily associated with the frontal region, with the highest proportion of connections occurring within the frontal lobe. Similarly, in the alpha band, no connections were related to the frontal lobe, as all were associated with either the parietal or temporal lobes. In the beta and gamma bands, all connections were related to the temporal regions. It is evident that the SEN connections were primarily located in regions of significant activity in the relative power spectrum.

**TABLE 2:** Percentage of connection counts in Figure 4a.

	Type		F‐F	F‐P	F‐O	F‐T	P‐P	P‐O	P‐T	O‐O	O‐T	T‐T
Delta	EC		62.2%	8.5%	—	26.8%	—	—	0.5%	—	—	2.0%
	EO		69.4%	19.2%	—	8.8%	0.5%	—	1.6%	—	—	0.5%
	EC vs. EO	PosT	—	—	—	55.0%	—	—	—	—	—	10.0%
		NegT	20.0%	15.0%	—	—	—	—	—	—	—	—
Theta	EC		57.9%	7.9%	—	30.8%	—	—	0.7%	—	—	2.7%
	EO		69.4%	18.8%	0.4%	11.0%	—	—	—	—	—	0.4%
	EC vs. EO	PosT	4.0%	—	—	—	—	—	—	—	—	4.0%
		NegT	64.0%	28.0%	—	—	—	—	—	—	—	—
Alpha	EC		—	—	—	—	48.4%	43.8%	0.5%	7.3%	—	—
	EO		—	3.1%	1.6%	45.0%	43.4%	1.1%	5.8%	—	—	—
	EC vs. EO	PosT	—	—	—	—	42.9%	35.7%	—	21.4%	—	—
		NegT	—	—	—	—	—	—	—	—	—	—
Beta	EC		—	1.9%	—	5.8%	5.8%	—	50.0%	—	3.9%	32.7%
	EO		—	—	—	6.5%	3.9%	2.6%	40.2%	—	15.6%	31.2%
	EC vs. EO	PosT	—	—	—	—	—	—	—	—	—	—
		NegT	—	—	—	100.0%	—	—	—	—	—	—
Gamma	EC		—	—	—	—	—	—	—	—	42.9%	57.1%
	EO		—	—	—	—	—	—	7.7%	—	38.5%	53.8%
	EC vs. EO	PosT	—	—	—	—	—	—	—	—	—	—
		NegT	—	—	—	—	—	—	—	—	—	100.0%

Abbreviations: F‐F, fronto‐frontal; F‐O, fronto‐occipital; F‐P, fronto ‐ parietal; F‐T, frontotemporal; NegT, Negative *t*‐test results; O‐O, occipito‐occipital; O‐T, occipito ‐ temporal; P‐O, parieto‐occipital; PosT, Positive *t*‐test results; P‐P, parieto‐parietal; P‐T, parieto ‐ temporal; T‐T, temporo‐temporal.

For the EO, the main SEN connections in the delta and theta bands were associated with the frontal lobe. Compared to the EC, the proportion of links related to the parietal lobe increased in EO. In the alpha band, the connections in the frontotemporal and parieto‐parietal regions both accounted for over 40%, while the parieto‐occipital connections in the EO decreased compared to the EC. The links in the beta and gamma bands were primarily associated with the temporal regions. Overall, SENs were predominantly located in brain regions exhibiting higher energy in the relative power spectrum.

The results of ESENA and the relative power spectrum for the EC versus EO are presented in Figure 4 and Table 2. In the delta band, the EC showed statistically significantly weaker SEN connections in the frontal region and the frontoparietal regions compared to the EO, which corresponds to the results from the power spectrum. However, EC exhibited significantly stronger SEN links between the frontal and temporal regions, which is inconsistent with the power spectrum results. In the theta band, the power spectrum analysis indicated no significant differences between the EC and the EO, while the ESENA results revealed significant differences in connections between the two states. Similarly, results from other frequency bands also demonstrated discrepancies between the ESENA results and the power spectrum analysis. Therefore, the ESENA may provide additional spatiotemporal information beyond the spatially focused relative power spectrum.

#### 3.2.3 Game‐Playing State vs. EC

**FIGURE 5:**
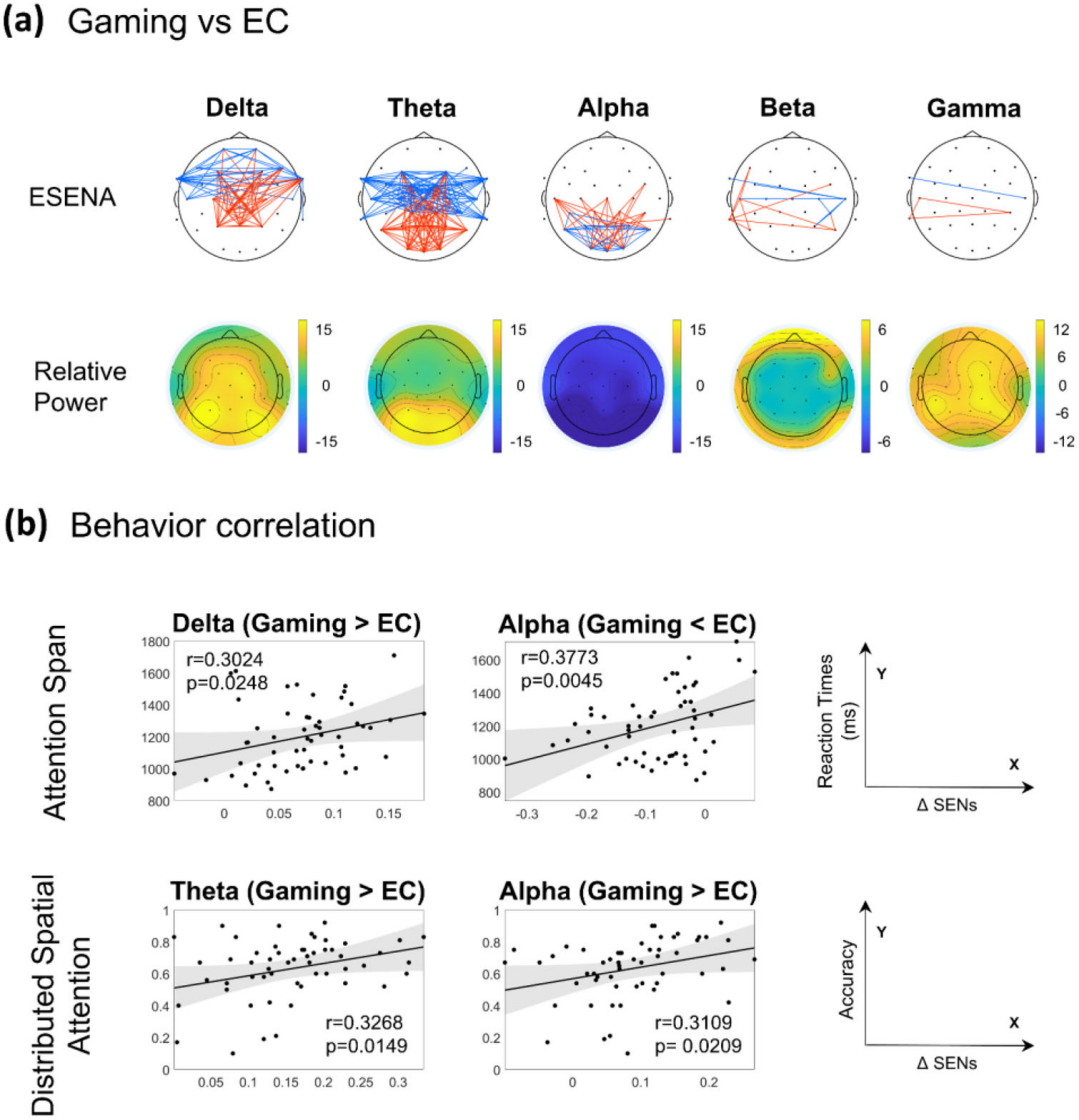
Results of the game‐playing state versus EC. (a) SENs and relative power of the game‐playing state versus EC (paired *t*‐test, FDR < 0.05; red: game > rest, blue: game < rest). (b) Correlations between the variations in the ΔSENs and reaction times in the attention span and distributed spatial attention tasks (*p* < 0.05). EC, eyes‐closed resting state; SENs, spatiotemporal event networks.

Figure 5a and Table 3 present the ESENA and relative power results for Dataset 3: the game‐playing state and EC are compared. In the delta band, ESENA indicated that stronger connections during the game‐playing state were observed between the midline frontal regions and between the frontal and parietal regions, which aligned with the results from the relative power spectrum. However, the significantly weaker SENs in the game‐playing state compared to EC were not reflected in the power spectrum. Similar to the delta band, the stronger regions in the game‐playing state were consistent with the power spectrum distribution for the theta band, while the weaker regions were not represented in the power spectrum. For the alpha band, the power spectrum results showed that the game‐playing state was significantly weaker than EC across the whole brain. However, the ESENA results were inconsistent with this: the weaker SENs in the game‐playing state were only distributed across the frontal and parietal regions, and there were also SENs where the gaming state was significantly stronger than EC. The SENs links in the beta and gamma bands were fewer, and, similarly, they were not entirely consistent with the power spectrum results.

**TABLE 3:** Percentage of connection counts in Figure 5a.

	Type		F‐F	F‐P	F‐O	F‐T	P‐P	P‐O	P‐T	O‐O	O‐T	T‐T
Delta	G vs. EC	PosT	22.1%	33.7%	—	—	7.8%	—	—	—	—	—
		NegT	13.0%	1.3%	—	19.5%	—	—	—	—	—	2.6%
Theta	G vs. EC	PosT	—	11.9%	11.9%	1.6%	7.1%	11.9%	3.2%	2.4%	2.4%	—
		NegT	13.5%	11.1%	—	16.0%	0.8%	—	4.0%	—	—	2.4%
Alpha	G vs. EC	PosT	2.4%	9.5%	2.4%	4.8%	16.7%	4.8%	—	7.1%	—	2.4%
		NegT	—	—	—	—	14.2%	19.0%	4.8%	7.1%	4.8%	—
Beta	G vs. EC	PosT	—	—	—	18.1%	—	—	27.3%	—	—	27.3%
		NegT	—	—	—	9.1%	—	—	9.1%	—	—	9.1%
Gamma	G vs. EC	PosT	—	—	—	—	—	—	50.0%	—	—	25.0%
		NegT	—	—	—	—	—	—	—	—	—	25.0%

Abbreviations: F‐F, fronto‐frontal; F‐O, fronto‐occipital; F‐P, fronto ‐ parietal; F‐T, fronto‐temporal; G versus EC, game‐playing state versus EC; NegT, Negative *t*‐test result; O‐O, occipito‐occipital; O‐T, occipito ‐ temporal; P‐O, parieto‐occipital; PosT, Positive *t*‐test result; P‐P, parieto‐parietal; P‐T, parieto ‐ temporal; T‐T, temporo‐temporal.

Figure 5b displays the correlations between the variations in the SENs (ΔSENs; game‐playing state SENs—EC SENs) and behavioral indicators in the attention span and distributed spatial attention tasks. Specifically, in the attention span test, the reaction times were found to correlate significantly with ΔSENs in the delta and alpha frequency bands. For the distributed spatial attention task, accuracy had notable correlations with the theta and alpha frequency bands.

## 4 Discussion

In this study, we introduced a method for mining spatiotemporal EEG information called ESENA. Contrasting with traditional methods, ESENA employs the strategy of mining frequently occurring synchronized spatiotemporal patterns. The method primarily examines the patterns of collaboration among different brain regions, focusing on mining information on stable inter‐regional connections under natural stimulation.

### 4.1 Methodology of ESENA

The brain's energy use can reflect neural activities, such as those in the primary motor cortex related to sensory feedback‐based motor control during movement and motor learning (Hamada et al. 2023). Currently, many EEG analysis methods, such as power spectrum (Z. Zhang 2019), microstate (Tarailis et al. 2023), and avalanche analyses (Arviv et al. 2015), are based on energy. In ESENA, to capture key and discrete events, regions with relative power exceeding that of the mean of the whole brain + 1 standard deviation were defined as activated regions. This definition of key events was also used previously for avalanche analysis (Arviv et al. 2015), and it can thus be flexibly applied according to the particular situation.

As a distributed and complex system, tight coordination between different regions of the brain is needed for the execution of its complex functions (Stark et al. 2008). For example, visual working memory requires the participation of modules including vision, memory, and attention (Olivers and Roelfsema 2020). Notably, brain activation patterns change in different stages of functional execution (Dayan and Cohen 2011; Sakai et al. 1998), and orderly changes in these patterns are crucial for functional realization (Braun et al. 2021). Furthermore, the sequence of brain activations differs between experts and nonexperts in some tasks (Anderson et al. 2016), which suggests that the order of brain activation may affect the efficiency of brain processing. Therefore, the sequence of brain activations is important for understanding functional implementation.

Currently, several methods have been developed for spatiotemporal pattern mining. The FOCA algorithm integrates temporal FC with spatial distribution consistency, providing a multidimensional assessment of the joint functional characteristics of voxels in image data (Dong, Luo, et al. 2014). The gSpan algorithm is primarily used to extract subgraph patterns in dynamic graphs, aiming to identify frequently occurring subgraph structures across different conditions and time periods from a set of dynamic graph networks (Iakovidou et al. 2013). The Apriori‐based time series pattern mining algorithm computes local correlations between time series using a sliding window, extracting frequent spatial object combinations from time series data (Atluri et al. 2014). However, these methods present certain limitations when applied to EEG data. They may be unsuitable for EEG data, struggle to address global spatiotemporal interactions, or focus primarily on frequent patterns within local time windows, neglecting the interactive dynamics between adjacent time windows. Furthermore, traditional DFC analysis primarily concentrates on the phase synchronization of spatially distinct regions, followed by the analysis of changes in phase synchronicity indices over time (Gschwandtner et al. 2021). However, it is regrettable that this approach focuses on real‐time synchronization of different regions, potentially overlooking pattern transitions that emerge over longer time scales. Therefore, ESENA focuses on co‐activation patterns across regions occurring in adjacent windows of the brain rather than real‐time co‐activation. Additionally, varying task demands might trigger different neural activation patterns, and sequences of sustained activation may represent key networks (Hardwick et al. 2013). Building upon the premise of sequentially activated connections over adjacent regions, ESENA highlights frequently occurring co‐activation patterns, which may represent core networks involved in brain activity. In brief, ESENA aims to mine information from frequently occurring sequential bursts of energy across adjacent brain regions, which may represent key sequences of brain activity.

### 4.2 ESENA Parameters

For parameter optimization, the key parameters of epoch length (corresponding to the Δ*t* in SEN's construction), event threshold, and data length were investigated. ICC values revealed instability in the delta, theta, and alpha bands for short epochs (≤ 4 s). However, beta and gamma rhythms displayed stable ICCs for segments > 3 s. This stability likely stems from the enhanced frequency resolution of longer epochs. While longer epochs improve resolution, they sacrifice temporal granularity. Furthermore, an assessment of event stability based on different epoch lengths and EEG data lengths (Figure S5) also indicated that most frequency bands tended to stabilize from 5‐s epochs onward. Based on the results of this study and previous research on brain networks and power spectrum calculations, we recommend using a 5‐s epoch (Jiao et al. 2023; Haartsen et al. 2020).

SVD, used for assessing event thresholds, revealed instability in the ESENA results across all frequency bands when the threshold was set before the mean + 1 standard deviation. Stability was attained by increasing the threshold to the mean + 1 standard deviation. It is noteworthy that high‐frequency bands consistently demonstrated greater stability compared with mid‐ and low‐frequency bands. The results from the EC shown in Figure S3 confirm that, at this latter threshold, the connection patterns gradually stabilized across all frequency bands. Additionally, it is evident that high‐frequency bands have fewer connections compared with low‐frequency bands. This may be because of the marked variability in the amplitude of low‐frequency oscillations and their widespread synchronous activity (Canolty and Knight 2010), which contrasts with high‐frequency oscillations—particularly beta and gamma waves—which exhibit less amplitude variability and are associated with localized brain activities (Maltez et al. 2004; Dustman et al. 1999). Using a lax threshold could lead to the mischaracterization of active connections, while a stringent threshold risks the omission of genuine connections and artificially enhanced network sparsity, potentially skewing subsequent analyses of outcomes. To circumvent these risks, we standardized the event threshold as the mean + 1 standard deviation.

The SVD analysis of ESENA data of different lengths revealed an initial decline in the first PC across all frequency bands, followed by stabilization. The decline is accelerated in lower frequency bands because of their inherently greater instability. Because of its nonstationary features (Lo et al. 2011), increasing EEG data length filters out unstable connections, thus accentuating stable ones. This effect is marked in low‐frequency bands, resulting in stabilization of the first PC across frequencies after 90 s.

### 4.3 SENs of EC

In Datasets 1 (Figure 3d), 2 (Figure 4), and 3 (Figure S6b), the EC relative power spectrum exhibits consistent results: the alpha band exhibits the highest energy, while the gamma band exhibits the lowest energy. Regarding the energy distribution, the delta and theta bands are predominantly in the frontal, parietal, and temporal lobes. Alpha energy is centered in the posterior parietal and occipital regions, with beta and gamma energies mainly being found in parietal and temporal regions, aligning with previous findings (Lee et al. 2023; Duan et al. 2021). The analysis of SENs for the EC across these datasets highlights distinct regional connections, including in the frontal and parietal lobes for delta and theta bands, the occipital lobe for the alpha band, and the regions for the beta and gamma bands, correlating with areas of high energy in the relative power spectrum and the recurrent activations observed in prior research (L. Zhang, Fan, et al. 2021; Frøkjær et al. 2017; Gibbings et al. 2021; Groppe et al. 2013). This consistent distribution demonstrates that SENs are distributed in regions associated with sustained brain activation. These preliminary findings confirm ESENA's proficiency in identifying brain regions with recurrent alternating energy bursts within time series on the basis of spatial energy distribution, as well as its potential to mine more complex brain information.

### 4.4 Differences in SENs Between the EC and EO

In the EC and EO, the ESENA connectivity analysis demonstrated a regional distribution that aligned with the relative power results, as depicted in Figure 4. Specifically, in the delta and theta bands, the EC was characterized by stronger connections associated with the frontal lobe. In contrast, the EO exhibited strong connections in both the frontal and parietal lobes in the theta band. This discrepancy might be attributable to a higher level of alertness during the EO (Gómez‐Ramírez et al. 2017). Such heightened alertness could lead to reduced activity in delta and theta bands in the frontal lobe, potentially explaining the more robust connectivity observed in the frontal lobe during the EC (Braboszcz and Delorme 2011; Johnstone et al. 2021; Bakry and Bakry 2019). Previous studies have suggested that, in the EO, there is significantly more visual sensory information processing and intensifying oscillations in theta bands in the frontal and parietal lobes (Petro et al. 2022). This accords with the enhanced connectivity observed between these lobes in these frequency bands during the EO. ESENA analysis also confirmed significant alpha‐band activity in the occipital lobe during the EC, consistent with previous findings (Hohaia et al. 2022). These results suggest that SENs not only reflect key energy distributions but also provide more comprehensive information than the relative power spectrum. This could be attributed to traditional power spectrum analysis primarily focusing on spatial energy distribution, overlooking temporal fluctuations in energy and the synergistic integration of the whole‐brain network. Consequently, ESENA has the potential to capture more comprehensive spatiotemporal patterns of brain activity.

### 4.5 SENs During Game‐Playing State

Figure 5a displays the results of ESENA and relative power spectra during the game‐playing state compared with the EC; the brain networks and DFC results are shown in Figures S6c and S7, respectively. According to these results, the primary differences between SENs and the relative power spectrum occur in the frontal area for the delta band, the temporoparietal links in the theta band, and the occipitoparietal connections in the alpha band. Previous studies have emphasized that networks involving frontal and parietal areas play a crucial role in the distribution of attention (Rossi et al. 2009). This enhanced attention allocation network strengthens the co‐activation patterns between frontal and parietal areas, explaining the stronger connections in these regions in SENs during the game‐playing state. Concurrently, heightened activation in frontal and parietal areas may lead to a reduction in their co‐activation with other parts of the brain, especially in the default network mode in the EC, which involves the frontal area and is believed to be negatively correlated with attention levels (Buckner and DiNicola 2019). This might explain the decreased frontal area connections in SENs during the game‐playing state. Additionally, the ΔSENs of the delta band were significantly correlated with reaction time in the attention span test, further demonstrating the efficacy of ESENA in extracting relevant information (Figure 5b).

In the theta band, the enhanced activation of the occipital and parietal lobes may be attributed to heightened attention, which is consistent with previous studies (Gong et al. 2019) and further corroborated by the results of the distributed spatial attention test (Figure 5b). The connectivity of SENs between the frontal and temporal lobes is reduced during the game‐playing state. This reduction in connectivity could be attributed to the stronger theta band activity in the default mode network, particularly within the frontal and medial temporal lobes, which decreases upon the redirection of attention to external stimuli (Cona et al. 2020).

Alpha‐mediated inhibition has been demonstrated to correlate with anticipatory attention in the visual domain. In a previous study, it was shown that, when observing various types of anticipated images, an increase in alpha power occurred in both the ventral and dorsal areas of the brain, indicating inhibitory effects during spatial attention (Foxe and Snyder 2011). The same effect was observed in the parietal lobe during selective auditory attention experiments (Kerlin et al. 2010). In the game‐playing state, players may have certain expectations and selectivity toward the images and sounds that appear. Repeated co‐activation in the occipital and parietal lobes of the brain is likely, which may account for the stronger connectivity observed between these lobes in the SENs in the game‐playing state. This correlation was also demonstrated in the attention span and spatial attention tests (Figure 5b). Notably, this was not reflected in the relative power spectrum or network.

In addition, although both DFC analysis and ESENA mine spatiotemporal information on the basis of networks, there exists a fundamental difference: the DFC method primarily focuses on the synchronicity of real signals (Guan et al. 2022), whereas ESENA chiefly uncovers alternating patterns of temporal activation. The DFC method was shown to be similar to the relative power spectrum and brain network analysis in this study (Figure S7). For example, stronger synchrony between the frontal and parietal lobes was observed in the delta band during the game‐playing state, and the EC demonstrated increased synchrony of the theta and alpha bands. These results indicate that ESENA analysis is capable of uncovering more information than traditional STIM methods.

### 4.6 Limitations

There are some limitations to our work. First, our method defines energy bursts occurring within a short time window as events and captures spatiotemporal characteristics on the basis of a network framework. However, as previously mentioned, the definition of events could be more flexible depending on practical needs. Second, the data segmentation was based on fixed time intervals. By employing this method, events occurring within these intervals were identified. However, it is important to note that brain activity does not typically follow a uniform time scale, such that our fixed interval approach could overlook complex patterns that do not fully conform to these strict time divisions (Nanda et al. 2023). To address this issue, future studies could consider incorporating variable time windows to more comprehensively capture the brain's dynamic neural activity. Finally, all aspects of this study involved the same population, and the results were not validated using datasets from other populations; this is important given that different populations may possess distinct baseline characteristics. Future research could consider adopting broader selection criteria to encompass a more diverse population, thereby enhancing the accuracy and reliability of the results.

## 5 Conclusions

This study introduces ESENA, a novel method for extracting spatiotemporal information from EEG data. The core principle of ESENA is to identify brain regions continuously activated at consecutive time points, reflecting changes in the brain's spatiotemporal patterns. ESENA has the potential to become an important tool in the field of neuroinformatics.

## Author Contributions

**Qiwei Dong**: data curation, formal analysis, methodology, software, writing–original draft, writing–review and editing, validation, visualization. **Runchen Yang**: formal analysis, software. **Xinrui Wang**: data curation, formal analysis. **Zongwen Feng**: formal analysis. **Chenggan Liu**: formal analysis, validation. **Shiyu Chen**: formal analysis. **Yuxi Zhou**: formal analysis. **Dezhong Yao**: conceptualization, funding acquisition. **Junru Ren**: validation. **Qi Xu**: conceptualization, supervision. **Li Dong**: conceptualization, methodology, supervision, resources, writing–review and editing, writing–original draft.

## Ethics Statement

The experiments were approved by the Ethics Committee of the Life Science and Technology Department, University of Electronic Science and Technology (Chengdu, China). All participants gave written informed consent.

## Conflicts of Interest

The authors declare no conflicts of interest.

### Peer Review

The peer review history for this article is available at https://publons.com/publon/10.1002/brb3.70426

## Supporting information



Supplementary Figure S1. Electrodes location. (a) 32‐channel system. (b) 64‐channel system

Supplementary Figure S2. ICC results of epoch selection. ICC, intraclass correlation coefficient.

Supplementary Figure S3. ESENA results of threshold selection (one sample *t*‐test, FDR < 0.05). M., mean; Std., standard deviation; ESENA, EEG Spatiotemporal Event Network Analysis

Supplementary Figure S4. ESENA results of data length selection (one sample *t*‐test, FDR < 0.05). ESENA, EEG Spatiotemporal Event Network Analysis.

Supplementary Figure S5. ESENA thresholds verification of different data lengths and epoch lengths (the red points are the 90 s data length threshold and 5 s epoch length threshold selected in this study). ESENA, EEG Spatiotemporal Event Network Analysis.

Supplementary Figure S6. ESENA and relative power results of EC and game‐playing state. (a) ESENA of game‐playing state and EC (one sample *t*‐test, FDR < 0.05). (b) Relative power results of game‐playing state and EC (one sample *t*‐test, FDR < 0.05). (c) Networks (using the Phase Synchronization Index method) results of game‐playing state and EC (one sample *t*‐test, FDR < 0.05). ESENA, EEG Spatiotemporal Event Network Analysis; EC, eyes‐closed resting state.

Supplementary Figure S7. DFC analysis results. (a) Left: EC's (PSI) 50 s time series example; right: 10 × 10 symmetric correlation matrices of 10 functional connectivity time series corresponding to four brain regions. Each position represents the Pearson correlation coefficient between two PSI time series. (b) left: 50 s time series example of game‐playing state; right: 4 brain regions of the game‐playing state corresponding to 10 FC time series 10 × 10 symmetric correlation matrixes. (c) The four brain regions of game‐playing state versus EC correspond to 10 functional connectivity time series *t* value matrices (paired sample *t*‐test, FDR < 0.05). (d) The difference between the DFC of game‐playing state and EC. Bar represents the frequency of occurrence of statistically significant DFC regions (game‐playing state vs. EC) within the scalp areas. DFC, dynamic functional connectivity; PSI, phase synchronization index; EC, eyes‐closed resting state; F, frontal lobe; T, temporal anterior; P, posterior lobe; O, occipital lobe.

Supplementary Figure S8. ESENA and relative power results of the full band (1–60 Hz). (a) ESENA results of EC, EO (one sample *t*‐test, FDR < 0.05), and EC versus EO (paired sample *t*‐test, FDR < 0.05). (b) ESENA results of game‐playing state, EC, and game‐playing state versus EC (one sample *t*‐test, FDR < 0.05). (c) Relative power of EC, EO (one sample *t*‐test, FDR < 0.05), and EC versus EO (paired sample *t*‐test, *p* < 0.01). (b) The relative power of the game‐playing state, EC, and game‐playing state versus EC (one sample *t*‐test, FDR < 0.05). ESENA, EEG Spatiotemporal Event Network Analysis; EC, eyes‐closed resting state; EO, eyes‐open resting state.

Supporting information

## Data Availability

The data that support the findings of this study are available from the corresponding author upon reasonable request, and the code will be posted on the GitHub website after the article is published.
