# Supplementary figures and images for: ESENA: A Novel Spatiotemporal Event Network Information Approach for Mining Scalp EEG Data
Source: Brain Behav. 2025 Mar 26;15(3):e70426. doi: 10.1002/brb3.70426 (PMC11937924; doi:10.1002/brb3.70426)

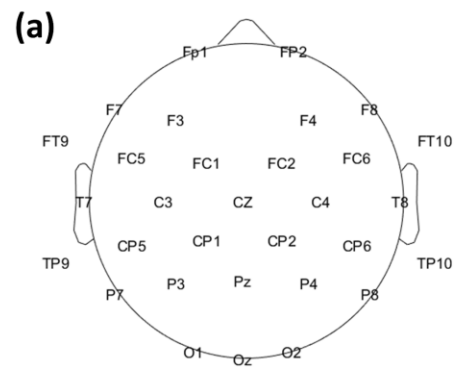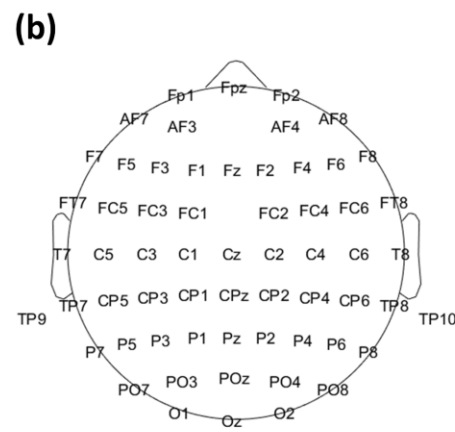

Supplementary Figure S1. Electrodes location. (a) 32-channel system. (b) 64-channel system

Supplement: Supplementary file 1 — Supplementary Figure S1. Electrodes location. (a) 32‐channel system. (b) 64‐channel system [file BRB3-15-e70426-s008.pdf]
