# Supplementary material for: ESENA: A Novel Spatiotemporal Event Network Information Approach for Mining Scalp EEG Data
Source: Brain Behav. 2025 Mar 26;15(3):e70426. doi: 10.1002/brb3.70426 (PMC11937924; doi:10.1002/brb3.70426)
Supplement: Supplementary file 2 — Supplementary Figure S2. ICC results of epoch selection. ICC, intraclass correlation coefficient. [file BRB3-15-e70426-s005.pdf]

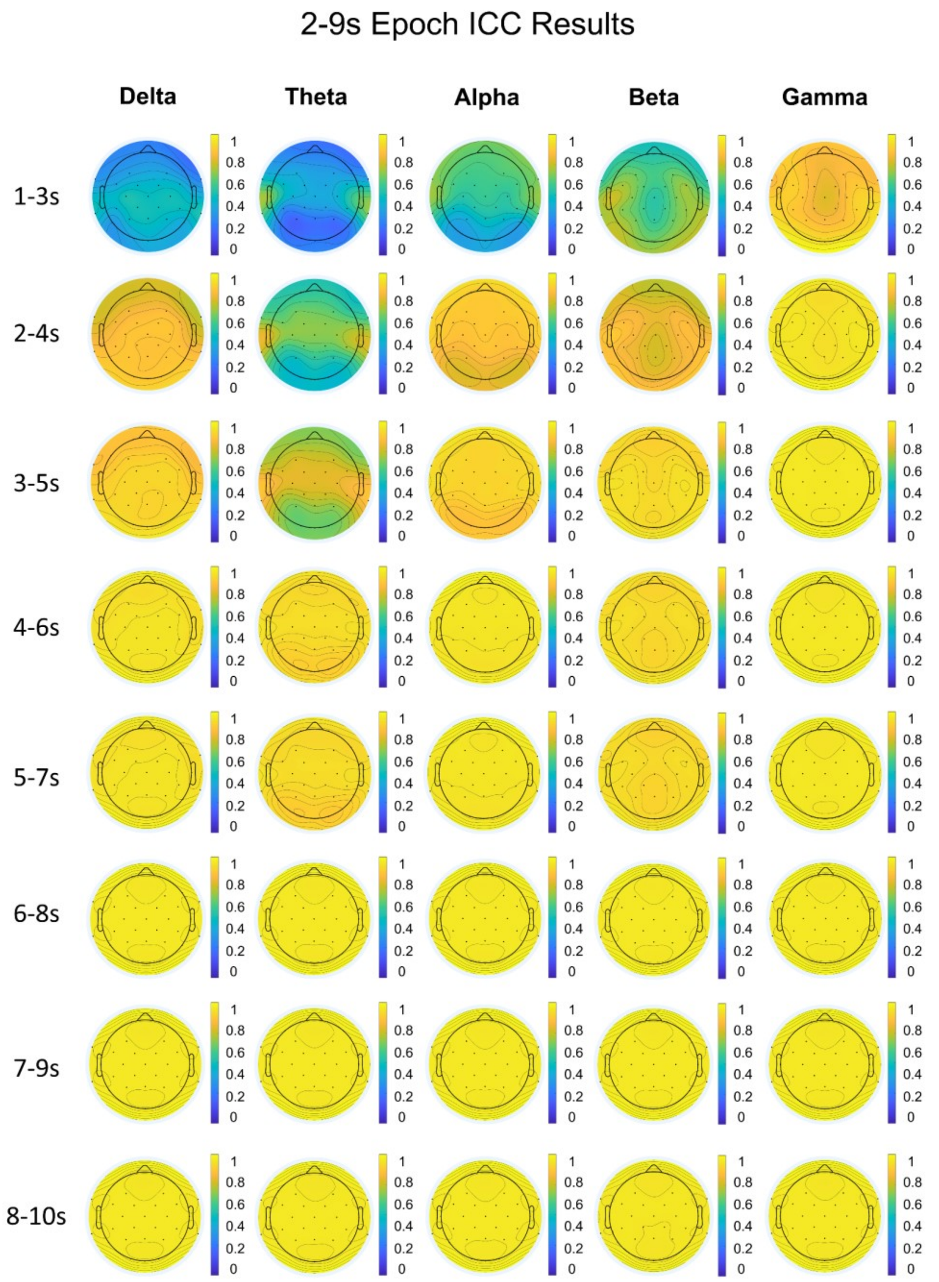

Supplementary Figure S2. ICC results of epoch selection. ICC, intraclass correlation coefficient.
