# Supplementary material for: ESENA: A Novel Spatiotemporal Event Network Information Approach for Mining Scalp EEG Data
Source: Brain Behav. 2025 Mar 26;15(3):e70426. doi: 10.1002/brb3.70426 (PMC11937924; doi:10.1002/brb3.70426)
Supplement: Supplementary file 3 — Supplementary Figure S3. ESENA results of threshold selection (one sample t‐test, FDR < 0.05). M., mean; Std., standard deviation; ESENA, EEG Spatiotemporal Event Network Analysis [file BRB3-15-e70426-s001.pdf]

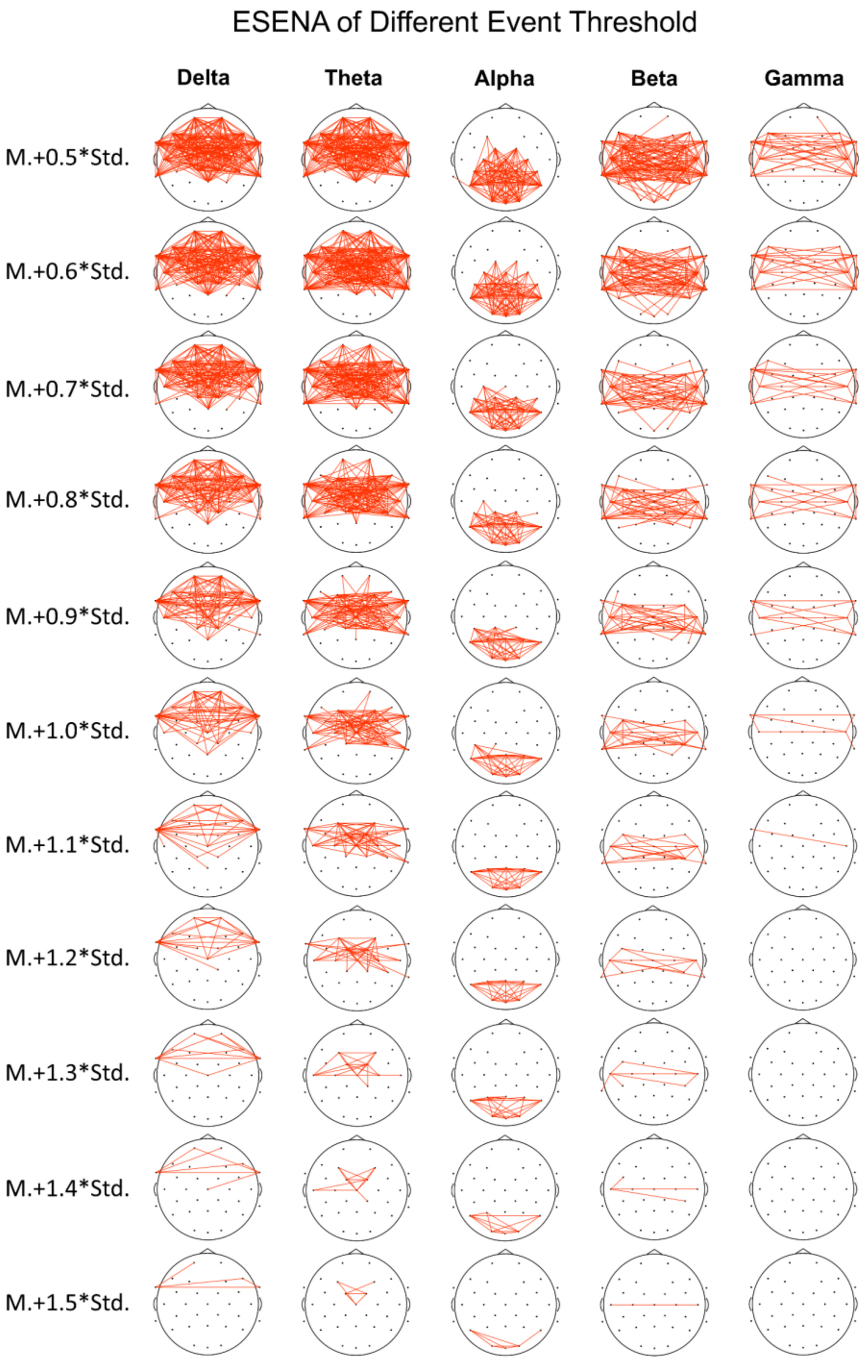

Supplementary Figure S3. ESENA results of threshold selection (one sample t-test, FDR<0.05). M., mean; Std., standard deviation; ESENA, EEG Spatio-temporal Event Network Analysis
