# Supplementary material for: ESENA: A Novel Spatiotemporal Event Network Information Approach for Mining Scalp EEG Data
Source: Brain Behav. 2025 Mar 26;15(3):e70426. doi: 10.1002/brb3.70426 (PMC11937924; doi:10.1002/brb3.70426)
Supplement: Supplementary file 4 — Supplementary Figure S4. ESENA results of data length selection (one sample t‐test, FDR < 0.05). ESENA, EEG Spatiotemporal Event Network Analysis. [file BRB3-15-e70426-s009.pdf]

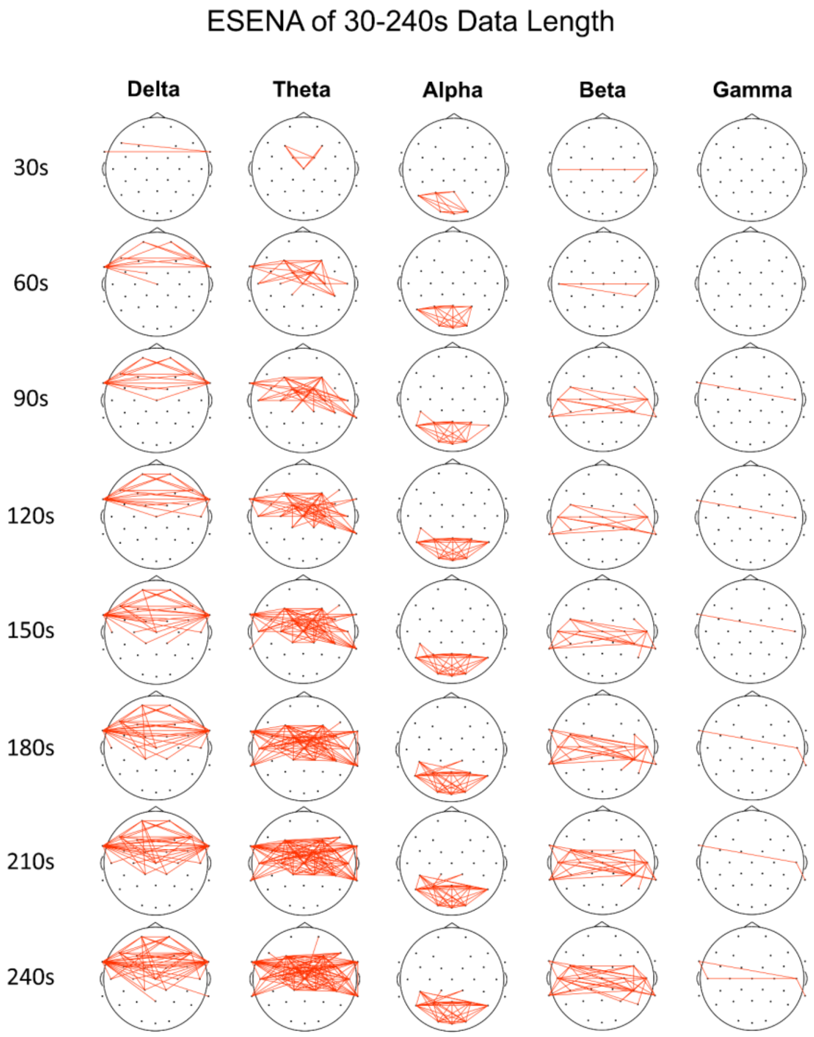

Supplementary Figure S4. ESENA results of data length selection (one sample t-test, FDR<0.05). ESENA, EEG Spatio-temporal Event Network Analysis.
