# Supplementary material for: ESENA: A Novel Spatiotemporal Event Network Information Approach for Mining Scalp EEG Data
Source: Brain Behav. 2025 Mar 26;15(3):e70426. doi: 10.1002/brb3.70426 (PMC11937924; doi:10.1002/brb3.70426)
Supplement: Supplementary file 5 — Supplementary Figure S5. ESENA thresholds verification of different data lengths and epoch lengths (the red points are the 90 s data length threshold and 5 s epoch length threshold selected in this study). ESENA, EEG Spatiotemporal Event Network Analysis. [file BRB3-15-e70426-s003.pdf]

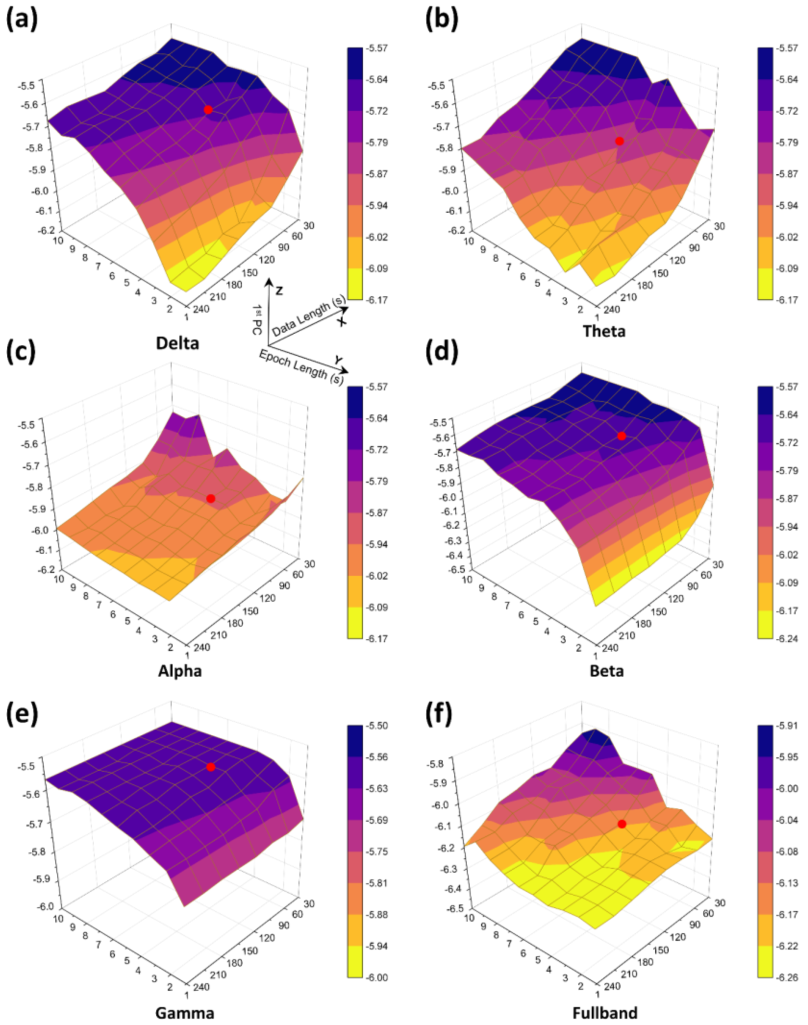

Supplementary Figure S5. ESENA thresholds verification of different data lengths and epoch lengths (the red points are the 90s data length threshold and 5s epoch length threshold selected in this study). ESENA, EEG Spatio-temporal Event Network Analysis.
