# Supplementary material for: ESENA: A Novel Spatiotemporal Event Network Information Approach for Mining Scalp EEG Data
Source: Brain Behav. 2025 Mar 26;15(3):e70426. doi: 10.1002/brb3.70426 (PMC11937924; doi:10.1002/brb3.70426)
Supplement: Supplementary file 6 — Supplementary Figure S6. ESENA and relative power results of EC and game‐playing state. (a) ESENA of game‐playing state and EC (one sample t‐test, FDR < 0.05). (b) Relative power results of game‐playing state and EC (one sample t‐test, FDR < 0.05). (c) Networks (using the Phase Synchronization Index method) results of game‐playing state and EC (one sample t‐test, FDR < 0.05). ESENA, EEG Spatiotemporal Event Network Analysis; EC, eyes‐closed resting state. [file BRB3-15-e70426-s007.pdf]

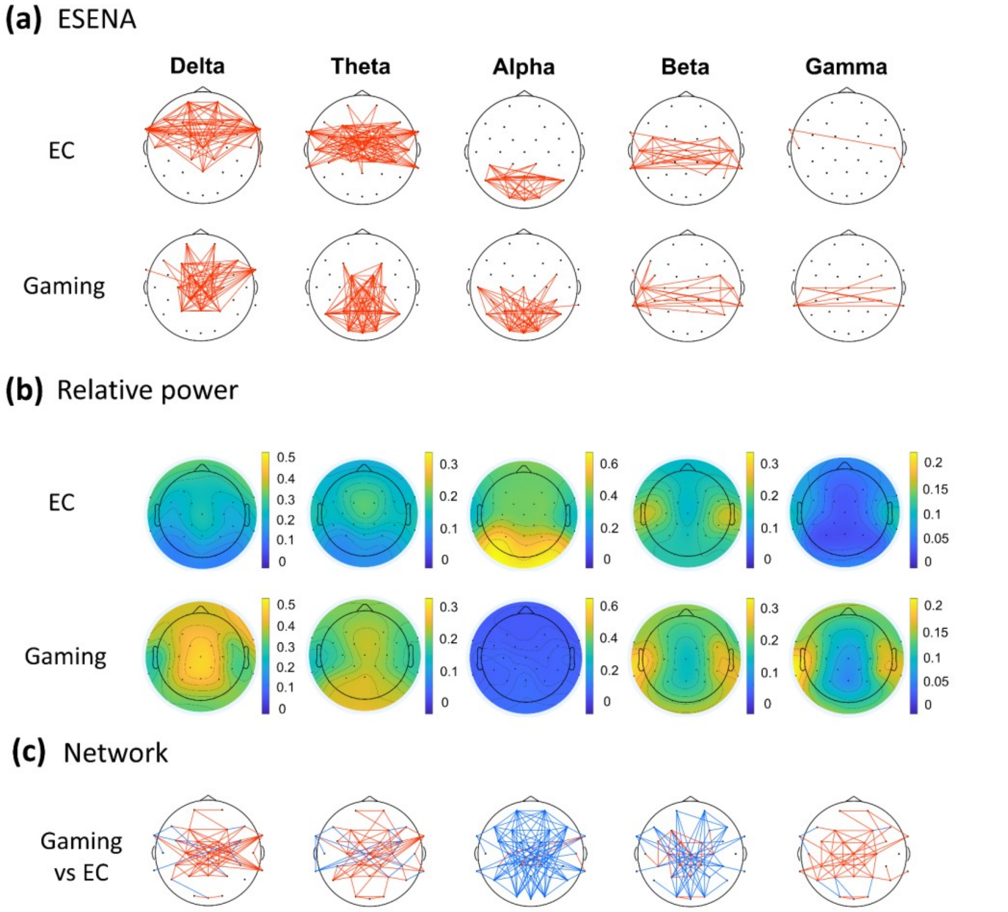

Supplementary Figure S6. ESENA and relative power results of EC and game-playing state. (a) ESENA of game-playing state and EC (one sample t-test, FDR<0.05). (b) Relative power results of game-playing state and EC (one sample t-test, FDR<0.05). (c) Networks (using the Phase Synchronization Index method) results of game-playing state and EC (one sample t-test, FDR<0.05). ESENA, EEG Spatio-temporal Event Network Analysis; EC, eyes-closed resting state.
