# Supplementary material for: ESENA: A Novel Spatiotemporal Event Network Information Approach for Mining Scalp EEG Data
Source: Brain Behav. 2025 Mar 26;15(3):e70426. doi: 10.1002/brb3.70426 (PMC11937924; doi:10.1002/brb3.70426)
Supplement: Supplementary file 7 — Supplementary Figure S7. DFC analysis results. (a) Left: EC's (PSI) 50 s time series example; right: 10 × 10 symmetric correlation matrices of 10 functional connectivity time series corresponding to four brain regions. Each position represents the Pearson correlation coefficient between two PSI time series. (b) left: 50 s time series example of game‐playing state; right: 4 brain regions of the game‐playing state corresponding to 10 FC time series 10 × 10 symmetric correlation matrixes. (c) The four brain regions of game‐playing state versus EC correspond to 10 functional connectivity time series t value matrices (paired sample t‐test, FDR < 0.05). (d) The difference between the DFC of game‐playing state and EC. Bar represents the frequency of occurrence of statistically significant DFC regions (game‐playing state vs. EC) within the scalp areas. DFC, dynamic functional connectivity; PSI, phase synchronization index; EC, eyes‐closed resting state; F, frontal lobe; T, temporal anterior; P, posterior lobe; O, occipital lobe. [file BRB3-15-e70426-s004.pdf]

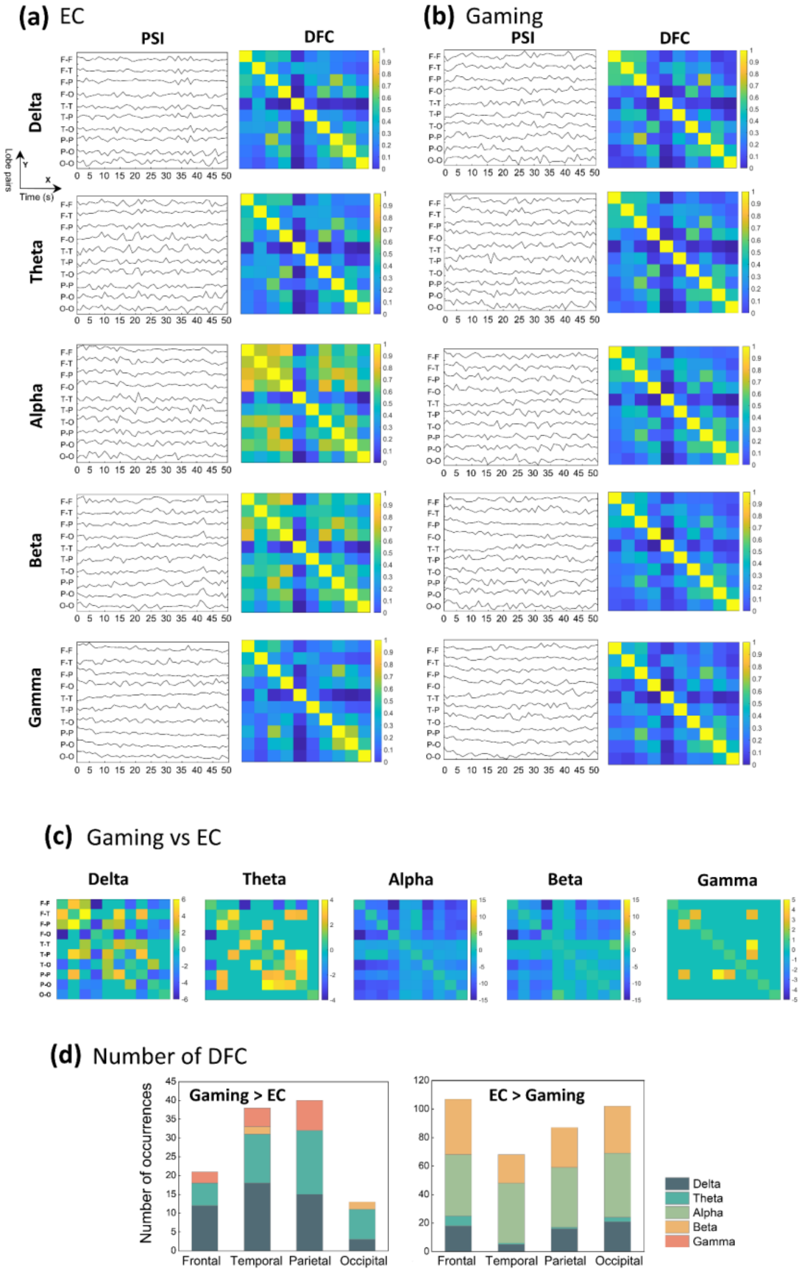

Supplementary Figure S7. DFC analysis results. (a) Left: EC's (PSI) 50s time series example; right: 10 × 10 symmetric correlation matrixes of 10 functional connectivity time series corresponding to 4 brain regions. Each position represents the Pearson correlation coefficient between two PSI time series. (b) left: 50s time series example of game-playing state; right: 4 brain regions of game-playing state corresponding to 10 functional connectivity time series 10 × 10 symmetric correlation matrixes. (c) The 4 brain regions of game-playing state vs EC correspond to 10 functional connectivity time series t-value matrices (paired sample t-test, FDR<0.05). (d) The difference between the DFC of game-playing state and EC. Bar represents the frequency of occurrence of statistically significant DFC regions (game-playing state vs EC) within the scalp areas. DFC, dynamic functional connectivity; PSI, phase synchronization index; EC, eyes-closed resting state; F, frontal lobe; T, temporal anterior; P, posterior lobe; O, occipital lobe.
