# Supplementary material for: ESENA: A Novel Spatiotemporal Event Network Information Approach for Mining Scalp EEG Data
Source: Brain Behav. 2025 Mar 26;15(3):e70426. doi: 10.1002/brb3.70426 (PMC11937924; doi:10.1002/brb3.70426)
Supplement: Supplementary file 8 — Supplementary Figure S8. ESENA and relative power results of the full band (1–60 Hz). (a) ESENA results of EC, EO (one sample t‐test, FDR < 0.05), and EC versus EO (paired sample t‐test, FDR < 0.05). (b) ESENA results of game‐playing state, EC, and game‐playing state versus EC (one sample t‐test, FDR < 0.05). (c) Relative power of EC, EO (one sample t‐test, FDR < 0.05), and EC versus EO (paired sample t‐test, p < 0.01). (b) The relative power of the game‐playing state, EC, and game‐playing state versus EC (one sample t‐test, FDR < 0.05). ESENA, EEG Spatiotemporal Event Network Analysis; EC, eyes‐closed resting state; EO, eyes‐open resting state. [file BRB3-15-e70426-s002.pdf]

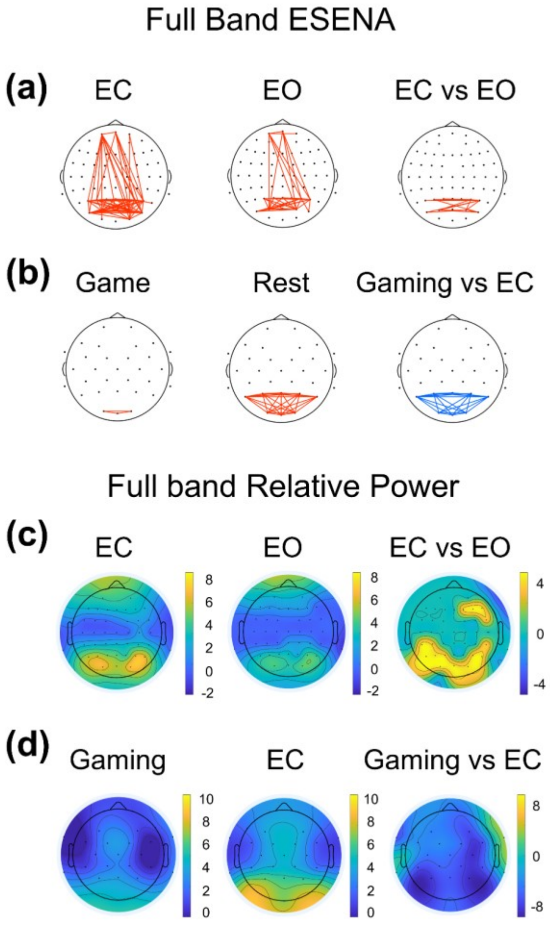

Supplementary Figure S8. ESENA and relative power results of the full band (1-60Hz). (a) ESENA results of EC, EO (one sample t-test, FDR<0.05) and EC vs EO (paired sample t-test, FDR<0.05). (b) ESENA results of game-playing state, EC, and game-playing state vs EC (one sample t-test, FDR<0.05). (c) Relative power of EC, EO (one sample t-test, FDR<0.05), and EC vs EO (paired sample t-test, p<0.01). (b) The relative power of game-playing state, EC, and game-playing state vs EC (one sample t-test, FDR<0.05). ESENA, EEG Spatio-temporal Event Network Analysis; EC, eyes-closed resting state; EO, eyes-open resting state.
