# Supplementary material for: ESENA: A Novel Spatiotemporal Event Network Information Approach for Mining Scalp EEG Data
Source: Brain Behav. 2025 Mar 26;15(3):e70426. doi: 10.1002/brb3.70426 (PMC11937924; doi:10.1002/brb3.70426)
Supplement: Supplementary file 9 — Supporting information [file BRB3-15-e70426-s006.pdf]

Supplementary Materials

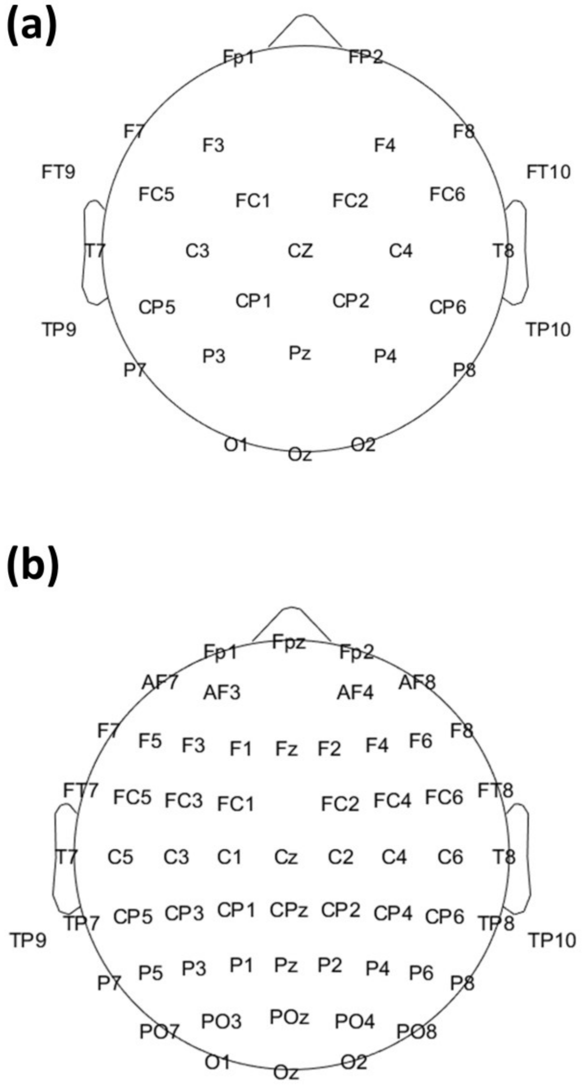

Supplementary Figure S1. Electrodes location. (a) 32-channel system. (b) 64-channel system

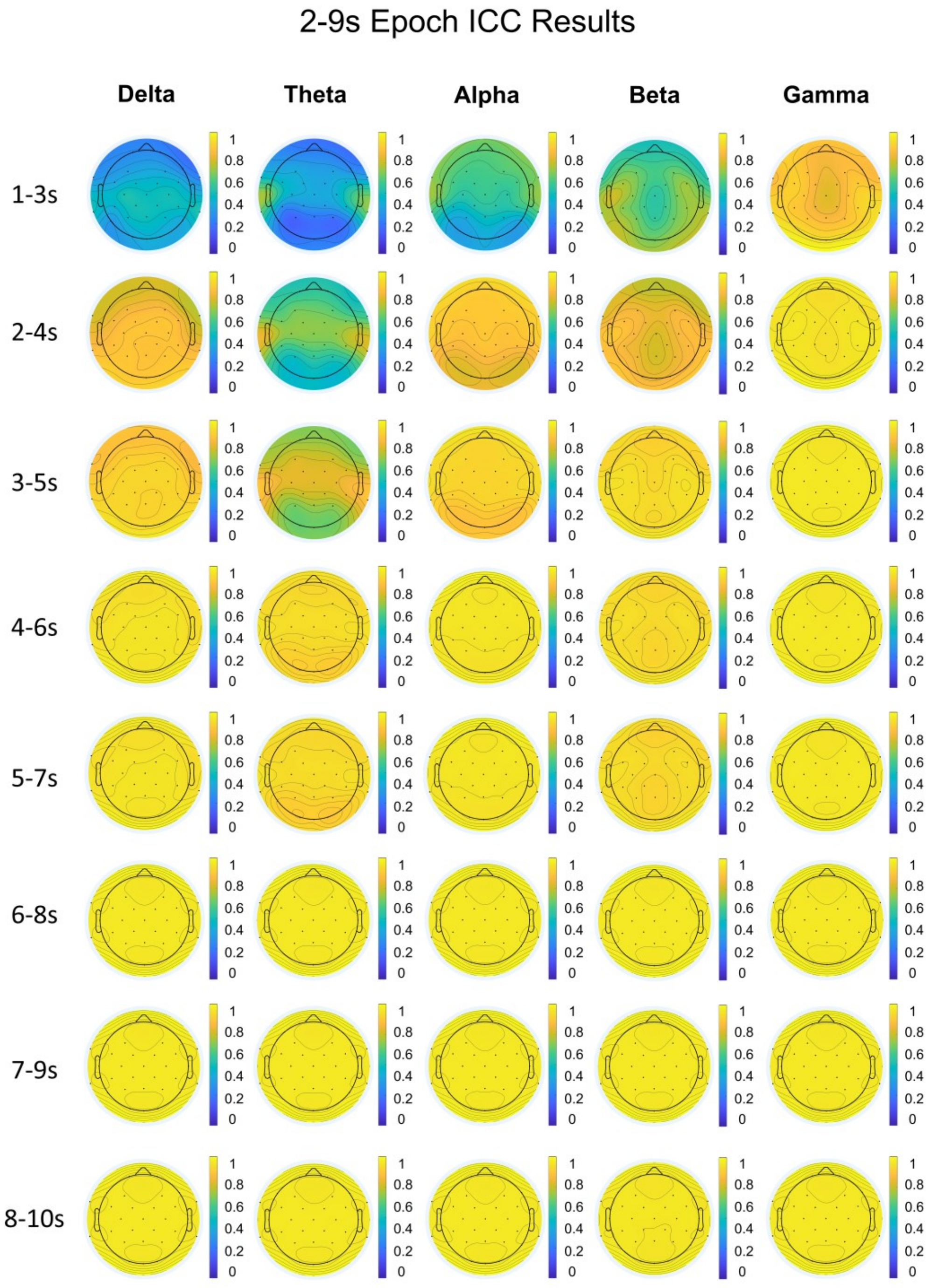

Supplementary Figure S2. ICC results of epoch selection. ICC, intraclass correlation coefficient.

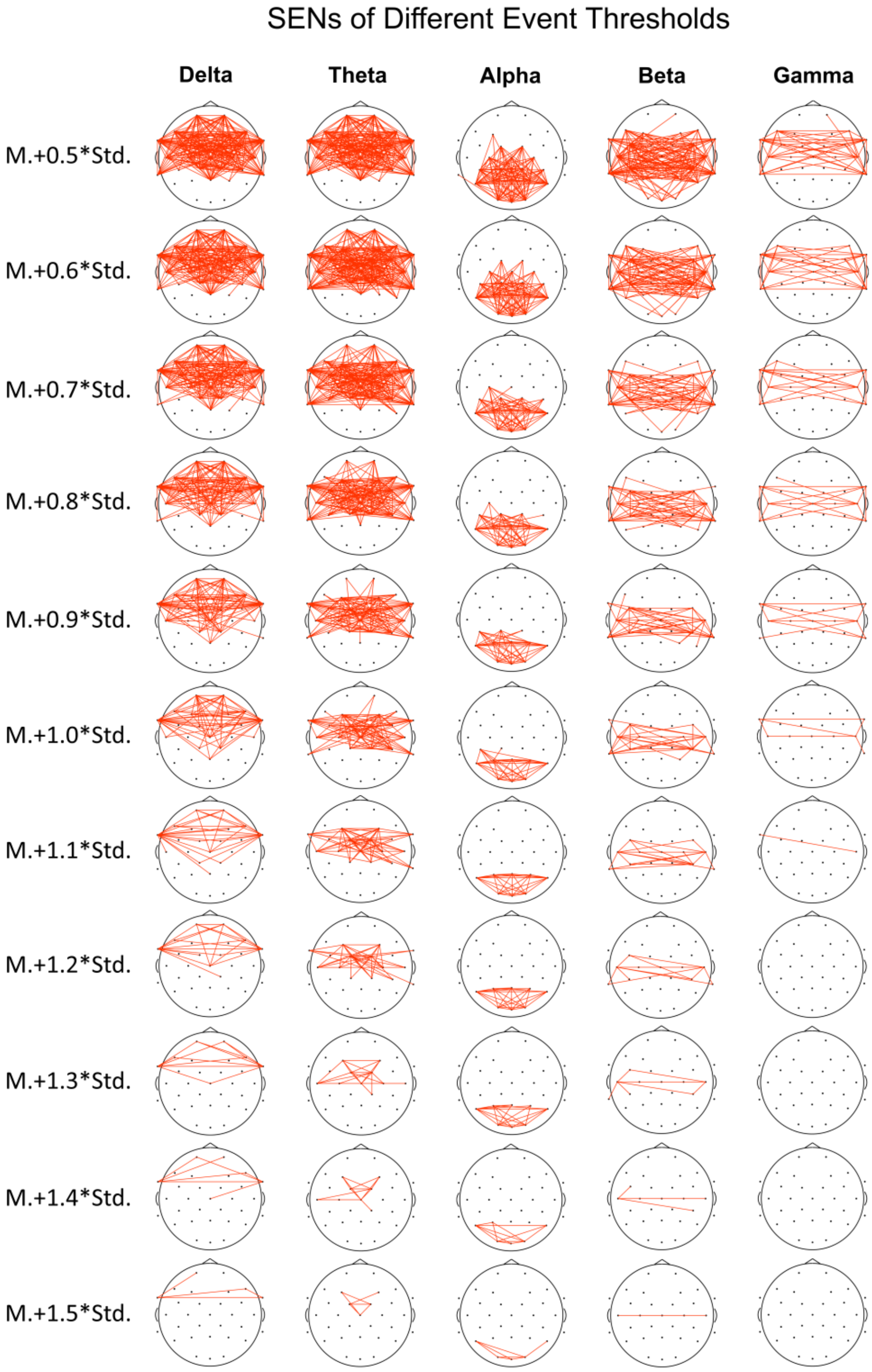

Supplementary Figure S3. ESENA results of threshold selection (one sample t-test, FDR<0.05). M., mean; Std., standard deviation; ESENA, EEG Spatio-temporal Event Network Analysis.

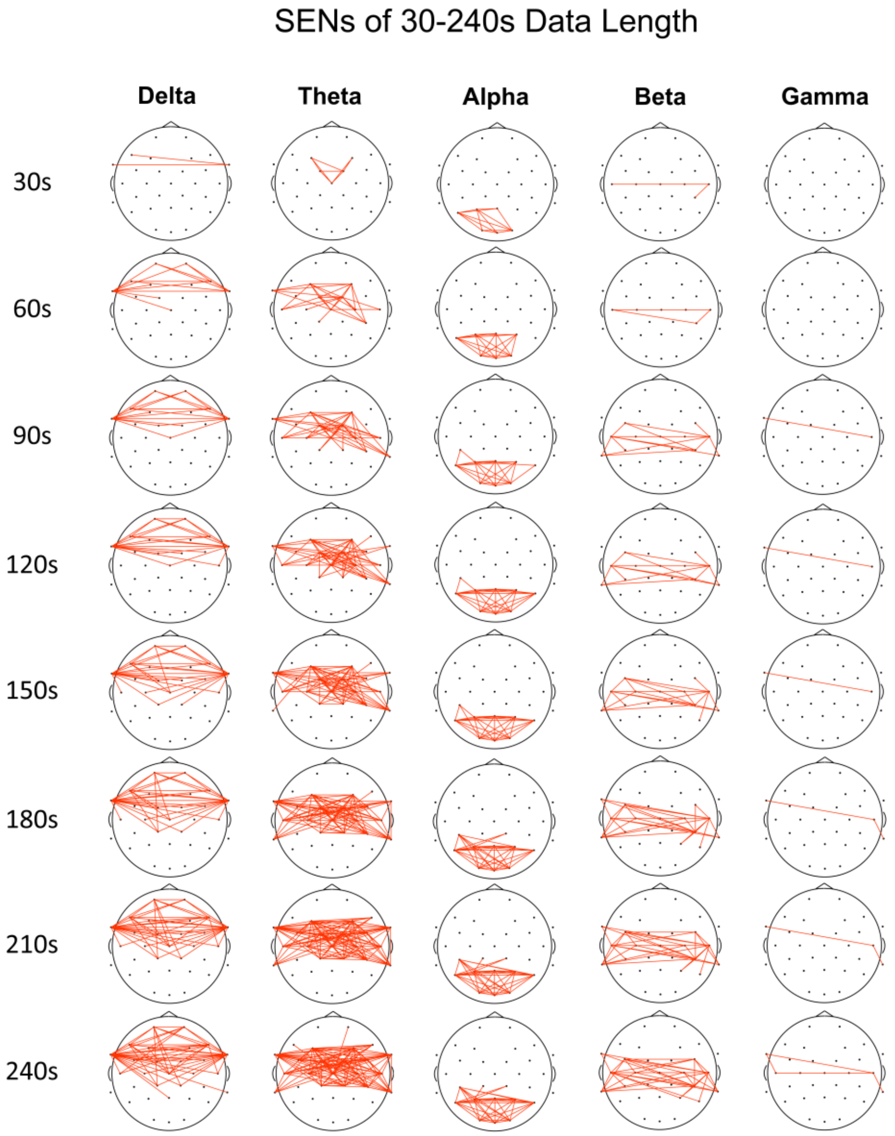

Supplementary Figure S4. ESENA results of data length selection (one sample t-test, FDR<0.05). ESENA, EEG Spatio-temporal Event Network Analysis.

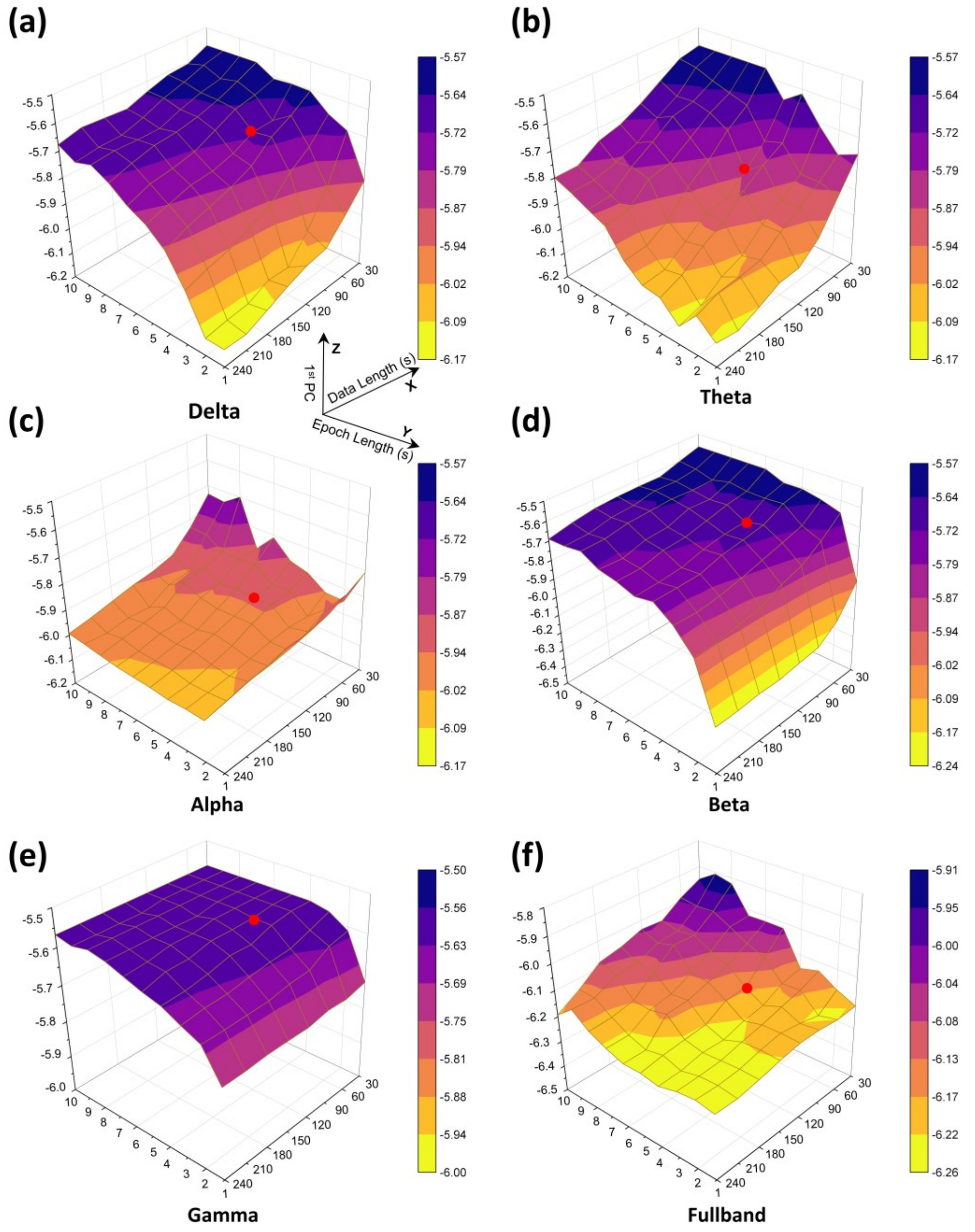

Supplementary Figure S5. ESENA thresholds verification of different data lengths and epoch lengths (the red points are the 90s data length threshold and 5s epoch length threshold selected in this study). ESENA, EEG Spatio-temporal Event Network Analysis.

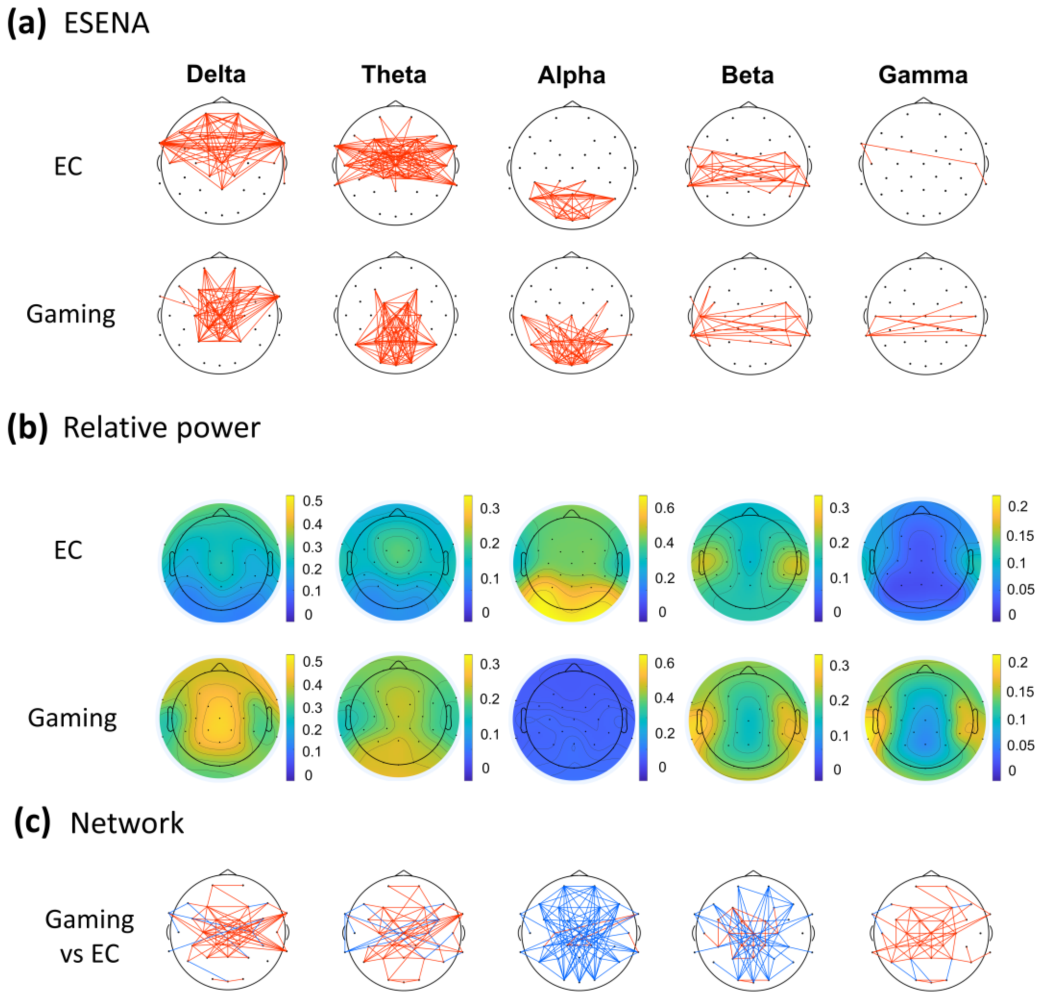

Supplementary Figure S6. ESENA and relative power results of EC and game-playing state. (a) ESENA of game-playing state and EC (one sample t-test, FDR<0.05). (b) Relative power results of game-playing state and EC (one sample t-test, FDR<0.05). (c) Networks (using the Phase Synchronization Index method) results of game-playing state and EC (one sample t-test, FDR<0.05). ESENA, EEG Spatio-temporal Event Network Analysis; EC, eyes-closed resting state.

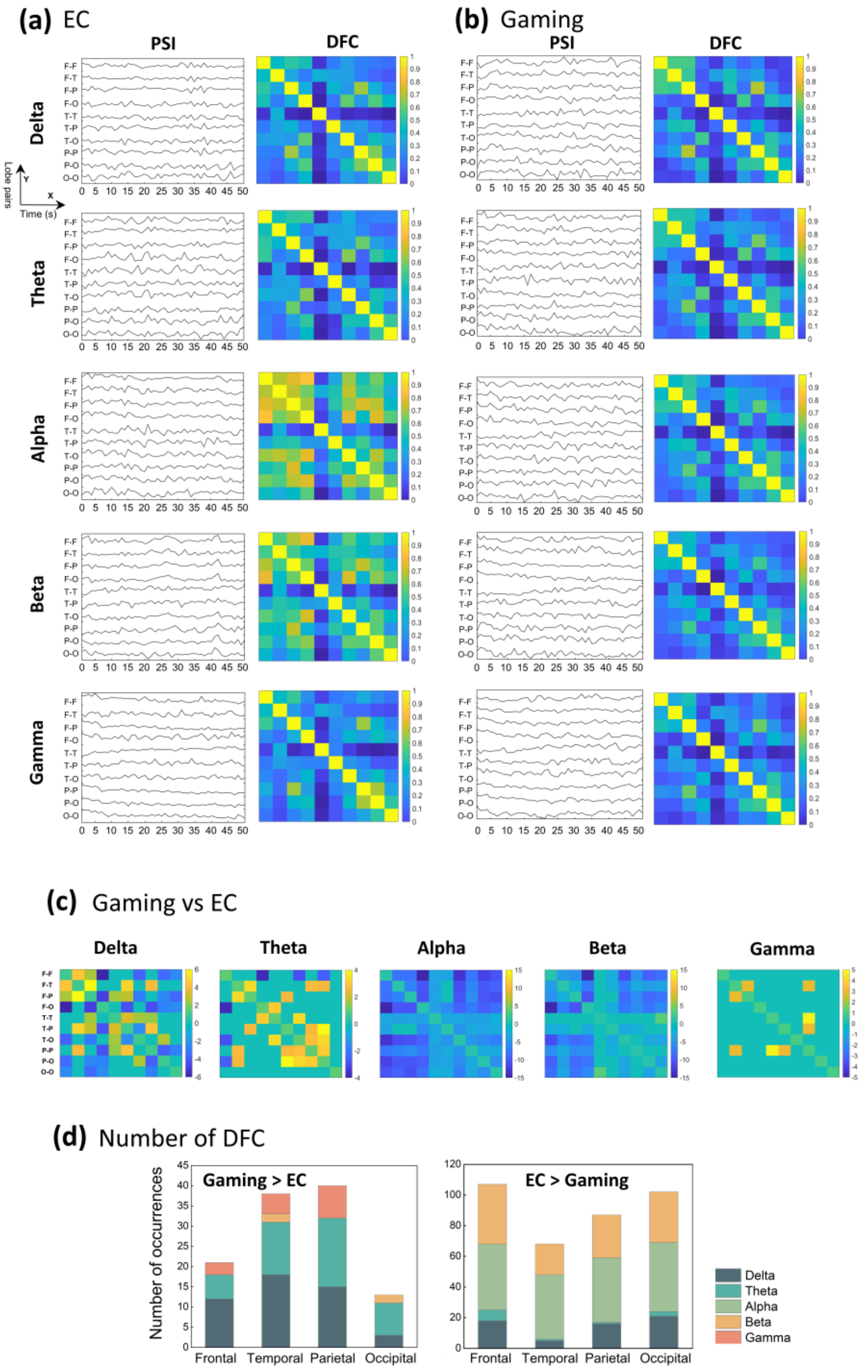

Supplementary Figure S7. DFC analysis results. (a) Left: EC's (PSI) 50s time series example; right: 10 × 10 symmetric correlation matrixes of 10 functional connectivity time series corresponding to 4 brain regions. Each position represents the Pearson correlation coefficient between two PSI time series. (b) left: 50s time series example of game-playing state; right: 4 brain regions of game-playing state corresponding to 10 functional connectivity time series 10 × 10 symmetric correlation matrixes. (c) The 4 brain regions of game-playing state vs EC correspond to 10 functional connectivity time series t-value matrices (paired sample t-test, FDR<0.05). (d) The difference between the DFC of game-playing state and EC. Bar represents the frequency of occurrence of statistically significant DFC regions (game-playing state vs EC) within the scalp areas. DFC, dynamic functional connectivity; PSI, phase synchronization index; EC, eyes-closed resting state; F, frontal lobe; T, temporal anterior; P, posterior lobe; O, occipital lobe.

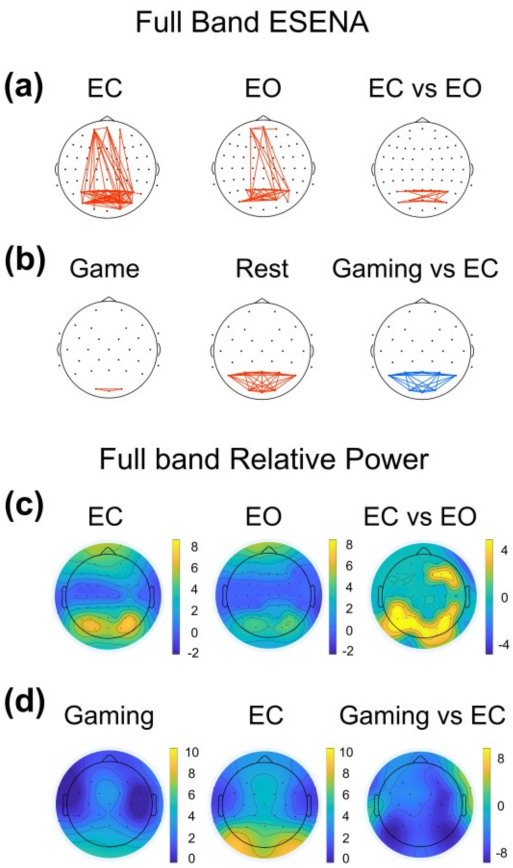

Supplementary Figure S8. ESENA and relative power results of the full band (1-60Hz). (a) ESENA results of EC, EO (one sample t-test, FDR<0.05) and EC vs EO (paired sample t-test, FDR<0.05). (b) ESENA results of game-playing state, EC, and game-playing state vs EC (one sample t-test, FDR<0.05). (c) Relative power of EC, EO (one sample t-test, FDR<0.05), and EC vs EO (paired sample t-test, p<0.01). (b) The relative power of game-playing state, EC, and game-playing state vs EC (one sample t-test, FDR<0.05). ESENA, EEG Spatio-temporal Event Network Analysis; EC, eyes-closed resting state; EO, eyes-open resting state.
